# Alteration of synonymous codon usage bias accompanies polyploidization in wheat

**DOI:** 10.3389/fgene.2022.979902

**Published:** 2022-10-14

**Authors:** Geng Tian, Guilian Xiao, Tong Wu, Junzhi Zhou, Wenjing Xu, Yanxia Wang, Guangmin Xia, Mengcheng Wang

**Affiliations:** ^1^ The Key Laboratory of Plant Development and Environment Adaptation Biology, Ministry of Education, School of Life Science, Shandong University, Qingdao, China; ^2^ Shijiazhuang Academy of Agriculture and Forestry Sciences, Shijiazhuang, China

**Keywords:** wheat, polyploidy, synonymous codon usage bias, nucleotide substitution, DNA methylation, epigenetic variation

## Abstract

The diploidization of polyploid genomes is accompanied by genomic variation, including synonymous nucleotide substitutions that may lead to synonymous codon usage bias (SCUB). SCUB can mirror the evolutionary specialization of plants, but its effect on the formation of polyploidies is not well documented. We explored this issue here with hexaploid wheat and its progenitors. Synonymous codons (SCs) ending in either cytosine (NNC) or guanidine (NNG) were more frequent than those ending in either adenosine (NNA) or thymine (NNT), and the preference for NNC/G codons followed the increase in genome ploidy. The ratios between NNC/G and NNA/T codons gradually decreased in genes with more introns, and the difference in these ratios between wheat and its progenitors diminished with increasing ploidy. SCUB frequencies were heterogeneous among exons, and the bias preferred to NNA/T in more internal exons, especially for genes with more exons; while the preference did not appear to associate with ploidy. The SCUB alteration of the progenitors was different during the formation of hexaploid wheat, so that SCUB was the homogeneous among A, B and D subgenomes. DNA methylation-mediated conversion from cytosine to thymine weakened following the increase of genome ploidy, coinciding with the stronger bias for NNC/G SCs in the genome as a function of ploidy, suggesting that SCUB contribute to the epigenetic variation in hexaploid wheat. The patterns in SCUB mirrored the formation of hexaploid wheat, which provides new insight into genome shock-induced genetic variation during polyploidization. SCs representing non-neutral synonymous mutations can be used for genetic dissection and improvement of agricultural traits of wheat and other polyploidies.

## Highlights

The bias for codons ending in cytosine and guanidine coincides with the decrease in DNA methylation-mediated conversion from cytosine to thymine during the formation of hexaploid wheat and possibly provides DNA methylation sites to promote epigenetic variation in the genome of hexaploid wheat. DNA methylation-mediated synonymous codon usage bias (SCUB) may account for the difference in genetic variation among the subgenomes of hexaploid wheat. The shift in SCUB demonstrates the bidirectional orchestration between genetic and epigenetic variation and mirrors the evolution process of polyploidies.

## Introduction

Polyploidization and whole-genome duplication are common evolutionary forces that have driven and shaped the evolution of all plants (Wendel 2000; Van de Peer et al., 2009; Jiao et al., 2011). The formation of polyploidies is followed by a whole-genome (or nearly so) diploidization process to produce viable progeny (Freeling et al., 2015; Van de Peer et al., 2017; Zhao et al., 2017; Cheng et al., 2018; Wendel et al., 2018). Diploidization is accomplished *via* large-scale genomic rearrangements (Chen and Ni 2006; Feldman and Levy 2012) that may induce a genome-wide genomic shock (Zohary and Feldman 1962; McClintock 1984). Genomic shock leads to diverse forms of genetic variation, among which nucleotide substitutions are the most common (Feldman and Levy 2012), and provide genetic diversity in polyploid species.

All amino acids except for methionine and tryptophan are encoded by at least two synonymous codons (SCs). SCs for the same amino acid display different frequencies in genomes, a phenomenon called synonymous codon usage bias (SCUB). Nucleotide substitutions in protein-coding sequences caused by natural variation change one codon into either a nonsynonymous codon or one of its SCs. Nucleotide substitution between SCs does not change the corresponding amino acid residue and is therefore often believed to be functionally neutral (King and Jukes 1969; Nei and Gojobori 1986). However, SCs affect recombination rates, splicing regulation, transcription efficiency, RNA secondary structure, mRNA stability, translational efficiency and accuracy in the regulation of gene expression, as well as protein folding (Marais et al., 2001; Warnecke and Hurst 2007; Zhang et al., 2009; Tuller et al., 2010; Presnyak et al., 2015). A recent report found that synonymous mutations in representative yeast genes are mostly strongly nonneutral (Shen et al., 2022). SCUB may therefore influence mutation rates, the extent of genetic drift and natural selection (Akashi and Eyre-Walker 1998; Akashi 2001; Guo and Yuan 2009; Wang et al., 2014), making it an important contributor to plant evolution. Given that widespread nucleotide substitutions follow the formation of polyploid individuals, it is an interesting issue that whether SCUB frequencies are different in polyploidies compared with their progenitors and mirror polyploidization events.

Intron gain and loss are key evolutionary forces of genomes (Knowles and McLysaght 2006; Sharpton et al., 2008; Tarrío et al., 2008) produced through transposon insertion (Giroux et al., 1994) or “reverse splicing” (Bonen and Vogel 2001) or as a by-product of errors during recombination (Mourier and Jeffares 2003). Insertion/deletion (InDel) events such as intron gain and loss necessarily entail the prior generation of DNA breaks and their repair, processes that are associated with genomic shock (Stoltzfus 2004; Rodríguez-Trelles et al., 2006) and may introduce local single-nucleotide polymorphisms (Tian et al., 2008; Chen et al., 2009; Choi et al., 2021). As a consequence of nucleotide substitutions, SCUB of exon sequences is related to adjacent introns in nuclear genomes (Hershberg and Petrov 2008). The propensity for intron gain or loss is associated with both intron number and intron position within the gene body (Coulombe-Huntington and Majewski 2007), so it is reasonable to speculate that SCUB may in turn also be related to these variables. Intron number and position have been proved to be associated with plant evolution (Qin et al., 2013). Whole-genome duplication affects intron characteristics such as alternative splicing (Iñiguez and Hernández 2017). However, the exact relationship between SCUB and intron number or position following polyploidization is unknown.

In addition to classical genetic variation, other sources of variation such as DNA methylation and changes in gene expression patterns are pervasive following interspecific hybridization and whole-genome duplications in allopolyploid species to cope with gene dosage (Adams 2007; Song and Chen 2015; Li et al., 2019). DNA methylation is also itself a major source of genomic variation, as methylated cytosine (5^m^C) is readily converted into thymine (Ossowski et al., 2010). Thus, DNA methylation-mediated conversion from cytosine to thymine may account for a fraction of SCUB events. However, the contribution of DNA methylation to SCUB in polyploid species has not been investigated. Moreover, epigenetic variation in polyploids is dynamic and reversible: for example, DNA methylation levels decreased in extracted tetraploid wheat derived from natural hexaploid wheat but increased in resynthesized hexaploid wheat from extracted tetraploid wheat (Yuan et al., 2020). However, any association between DNA methylation and the extent of SCUB, especially the role of SCUB in DNA methylation changes, has not been reported in polyploid species.

Hexaploid bread wheat (*Triticum aestivum* L.) arose from two rounds of interspecific hybridization and whole-genome duplication (Salamini et al., 2002; Bevan et al., 2017). The formation of tetraploid wheat (*Triticum durum*, AABB) entailed the hybridization of the A subgenome progenitor red wild einkorn wheat (*Triticum urartu*) and an unknown B subgenome progenitor, followed by a second hybridization event leading to hexaploid wheat (AABBDD) between tetraploid wheat and the D subgenome progenitor rough-spike hard grass (*Aegilops tauschii*). In this study, we used hexaploid wheat and its tetraploid and diploid progenitors as test cases and described the effect of polyploidization on SCUB and the associated close link between DNA methylation and SCUB.

## Results

### SCUB patterns differ between hexaploid wheat and its progenitors

The three stop codons had lower frequencies than those encoding amino acids, and the proportions of amino acid encoding codons represented between 0.43% (CGA in *T. aestivum*) and 3.92% (GAG in *T. aestivum*). In addition, amino acid-specifying codons followed similar patterns in hexaploid wheat and its progenitor species, although the A subgenome diploid progenitor *T. urartu* was slightly distinct from the other species (Supplementary Figure S1). We used 59 SCs for further analysis by excluding the unique codons ATG (for Met) and TGG (for Trp). Relative synonymous codon usage (RSCU) values for these 59 SCs varied from 0.35 (TTA in *T. aestivum*) to 1.77 (CTC in *T. aestivum*) in the *Triticum/Aegilops* spp.; RSCU values for most SCs in *T. urartu* were quite different from those of other species, and the coefficients of variation (CVs) of RSCU values between hexaploid wheat and its progenitors decreased when *T. urartu* was not included in the calculation, as with SC frequencies (Supplementary Table S1).

**FIGURE 1:**
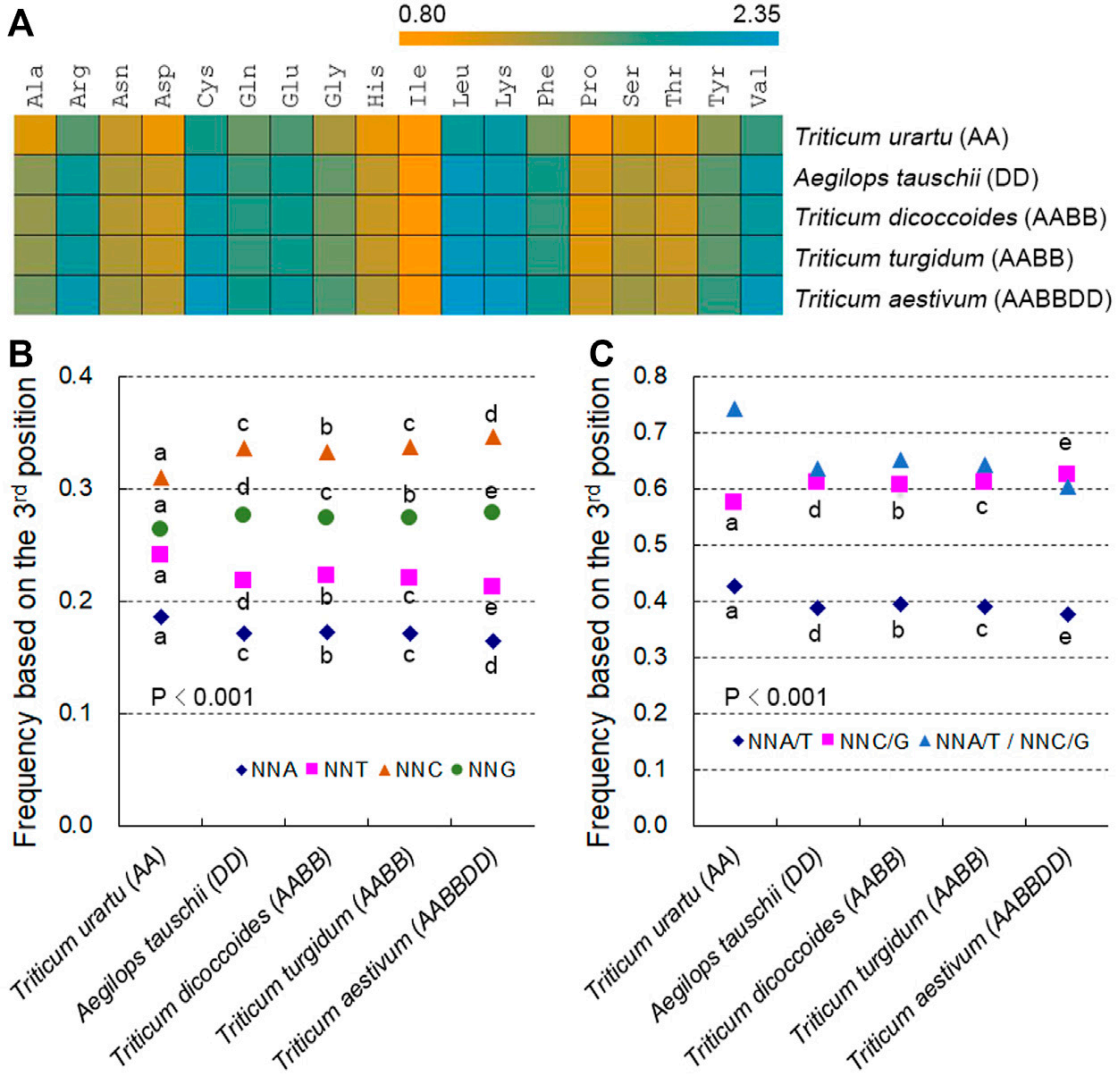
SCUB is heterogeneous between hexaploid wheat and its progenitors. **(A)** Ratio between the numbers of C/G-ending SCs and of A/T-ending SCs for 18 amino acids (Met and Trp not included). **(B)** Frequency of NNA, NNT, NNC and NNG codons. NNA, NNT, NNC and NNG: SCs with A, T, C and G as the final base, respectively; N denotes any base. The frequency was calculated as the ratio between the number of all SCs ending with A, T, C or G and the total number of SCs. **(C)** Frequency of NNA/T and NNC/G codons. NNA/T and NNC/G: SCs with A and T or C and G as the final base, respectively; N denotes any base. The frequency was calculated as the ratio between the number of all SCs ending with A and T or C and G and the total number of SCs. Statistical comparison was conducted by Chi square (χ^2^) test; the difference between two species was calculated with Chi square partitioning; different lowercase letters represent significantly different values (*p* < 0.05).

Among the 59 SCs specifying 18 amino acids, codons ending in C or G (NNC/Gs) were more frequent than those ending in A or T (NNA/Ts) (Supplementary Table S1). The frequencies of 59 SCs were strongly and positively correlated with RSCU values between species (*r* > 0.827, except for four codons whose RSCU and SCUB values were almost identical across *Triticum/Aegilops* spp.) (Supplementary Table S1; Supplementary Figure S2). To gain a more direct view of SCUB, we defined SCUB frequency of a given amino acid encoded by synonymous codons (SCs) as the ratio of the number of NNC/Gs to that of NNA/Ts (Figure 1A). SCUB frequencies for individual amino acids ranged from 0.808 (Ile in *T. urartu*) to 2.335 (Leu in *T. aestivum*) (Figure 1A). With the exception of Ile, SCUB frequencies of other amino acids were all higher than 1, indicative of the bias to NNC/Gs in hexaploid wheat and its progenitors. SCUB frequencies also showed a difference between hexaploid wheat and its progenitors, among which the A subgenome diploid progenitor *T. urartu* exhibited the lowest frequencies and hexaploid wheat the highest; the AB subgenome tetraploid progenitors *T. dicoccoides* and *T. turgidum* had moderate frequencies, while the difference between *T. dicoccoides* and *T. turgidum* was quite small (Supplementary Table S2). Moreover, the frequencies of the D subgenome progenitor *A. tauschii* were also lower than those of hexaploid wheat.

SCUB was further directly reflected in the total SCUB frequencies of NNA, NNT, NNC and NNG codons, which were each calculated as the ratio between the number of all NNA, NNT, NNC or NNG codons across all 59 SCs and the number of 59 SCs for all coding regions in the genome under consideration. NNCs and NNGs were more abundant than NNAs and NNTs in *Triticum/Aegilops* spp., with NNCs being more frequent than NNGs and NNAs being less frequent than NNTs (Figure 1B; *p* < 0.001, χ^2^ test). The frequencies of NNC/Gs were higher than those of NNA/Ts, and the ratios between NNA/Ts and NNC/Gs were below 1 (Figure 1C). The frequencies of NNA, NNT, NNC or NNG codons differed between hexaploid wheat and its diploid and tetraploid progenitors (Figure 1B; Supplementary Table S3). The frequencies of NNAs and NNTs were the highest in the A subgenome diploid progenitor *T. urartu*, the lowest in hexaploid wheat, and intermediate in the AB subgenome tetraploid progenitors *T. dicoccoides* and *T. turgidum*; the frequencies of NNCs and NNGs exhibited the opposite pattern. *T. urartu* had the highest NNA/T frequencies of all genomes analyzed but also the lowest NNC/G frequencies, with hexaploid wheat having the lowest NNA/T frequencies and the highest NNC/G frequencies, and *T. dicoccoides* and *T. turgidum* intermediate NNA/T and NNC/G frequencies (Figure 1C; Supplementary Table S3). The ratio between NNA/Ts and NNC/Gs was therefore up to 0.743 in *T. urartu*, was low to 0.603 in hexaploid wheat, and was around 0.650 in *T. dicoccoides* and *T. turgidum*. Moreover, the D subgenome progenitor diploid *A. tauschii* also displayed higher NNA, NNT and NNA/T frequencies but lower NNC, NNG and NNC/G frequencies than hexaploid wheat (Figures 1B,C; Supplementary Table S3). Of the two tetraploid progenitors, *T. dicoccoides* had higher NNA, NNT and NNA/T frequencies but lower NNC, NNG and NNC/G frequencies than *T. turgidum*, although this difference was not as pronounced as those between tetraploid and diploid/hexaploid species (Figures 1B,C; Supplementary Table S3). The difference in codon adaptation index (CAI) and other indices among *Triticum/Aegilops* spp. Also globally agreed with total SCUB frequencies (Supplementary Table S4), demonstrating that SCUB frequencies can reflect SCUB characteristics and differences between hexaploid wheat and its progenitors.

We further compared the difference in SCUB among subgenomes, and found that NNC, NNG and NNC/G frequencies were higher than NNA, NNT and NNA/T frequencies in each of subgenomes of tetraploid and hexaploid wheat (Supplementary Figure S3). As for A, B or D subgenome, following the rise of genome ploidy, NNC, NNG and NNC/G frequencies gradually increased, while NNA, NNT and NNA/T frequencies gradually decreased (Supplementary Figure S3B), alike the difference based on the whole genome. However, NNA/T to NNC/G ratios of A subgenome were lower than those of B subgenome in either tetraploid or hexaploid wheat (*p* = 1.01 × 10^−190^–7.58 × 10^−37^), but the ratio of A subgenome were higher than that of D subgenome in hexaploid wheat (*p* = 1.74 × 10^−15^) (Supplementary Figure S3A). More importantly, NNA/T to NNC/G ratios were different between diploid *T. urartu* and *A. tauschii*, but they were similar between A and D subgenomes in hexaploid wheat, showing the trend to homogeneity of SCUB among subgenomes in polyploid wheat.

### SCUB increases linearly with the rise in intron number

**FIGURE 2:**
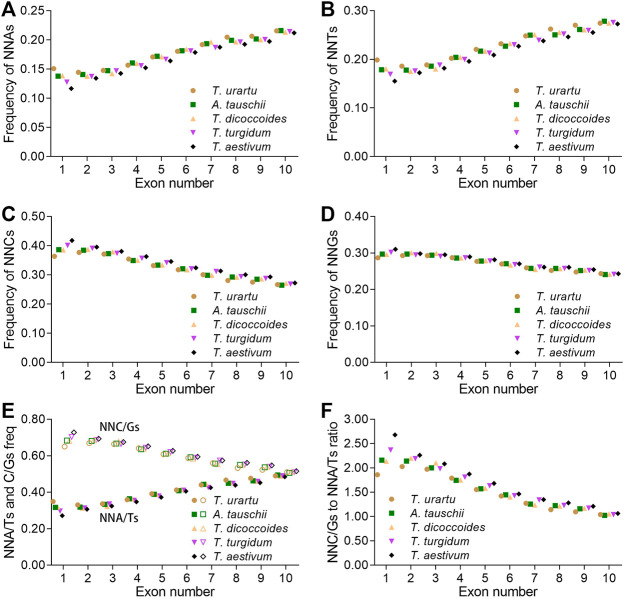
Influence of the number of introns on SCUB. Frequencies of A-ending SCs (NNAs) **(A)**, T-ending SCs (NNTs) **(B)**, C-ending SCs (NNCs) **(C)**, G-ending SCs (NNGs) **(D)**, or A/T- and C/G-ending SCs (NNA/Ts and NNC/Gs) **(E)** in genes with up to nine introns. **(F)** Ratios between A/T-ending SCs and C/G-ending SCs (NNA/Ts and NNC/Gs) in genes with up to nine introns. N denotes any base. The difference between hexaploid wheat and its progenitors was calculated by Chi square (χ^2^) test, and the results are presented in Supplementary Table S5.

SCUB is associated with plant evolution (Qin et al., 2013). To assess whether SCUB frequency was associated with the number of introns in the formation of polyploid wheat, we compared SCUB frequencies between genes with different number of exons. Within *Triticum/Aegilops* spp., the frequencies of NNA and NNT gradually increased with increasing exon number, while the frequencies of NNC and NNG gradually decreased (Figures 2A–D; Supplementary Figure S4). We noticed an exception with genes containing one or two exons in *T. urartu* and *T. dicoccoides.* In genes with fewer than ten exons, the frequencies of NNC/G were higher than those of NNA/T (*p* = 5.12 × 10^−7^ ∼ 6.87 × 10^−4^, *t*-test). For genes with exactly ten exons, the difference between NNA/T and NNC/G frequencies remained significant but weaker (*p* = 0.003) (Figures 2E,F).

The A subgenome diploid progenitor *T. urartu* had the highest NNA, NNT and NNA/T frequencies in genes with one to ten exons, hexaploid wheat had the lowest, and tetraploid *T. dicoccoides* and *T. turgidum* had intermediate frequencies; the frequencies of NNCs, NNGs, and NNC/Gs followed opposite patterns, resulting in the highest NNC/G to NNA/T ratios in hexaploid wheat but the lowest in *T. urartu* (Figure 2; Supplementary Table S5). As with other progenitors, the D subgenome diploid progenitor *A. tauschii* had higher NNA, NNT and NNA/T frequencies but lower NNC, NNG and NNC/G frequencies than hexaploid wheat. The difference in SCUB frequencies between hexaploid wheat and its progenitors was the most pronounced in genes with one exon but became gradually smaller as exon number rose from two to ten, especially in genes with ten exons (Figure 2; Supplementary Table S5). For instance, the CV value for NNA frequency was 0.096 in genes with one exon, 0.011–0.028 for genes with two to nine exons, and 0.008 in genes with ten exons. Moreover, the difference in NNA, NNT, NNC and NNG codons between hexaploid wheat and its progenitors varied, with NNG frequencies in genes with one to ten exons being smaller than the frequencies of other codons.

The SCUB patterns based on exon number among the subgenomes were similar, and the NNC/G to NNA/T ratios gradually decreased following the rise of exon number (Supplementary Figure S5). The NNC/G to NNA/T ratios among the subgenomes were different from each other in genes with less exons in either tetraploid or hexaploid wheat, and the difference became smaller in genes with more exons (Supplementary Figure S5A–C). On the other hand, for A, B or D subgenome, the NNC/G to NNA/T ratios showed different among hexaploid wheat and its progenitors, and the ratios increased with the rise of genome ploidy (Supplementary Figure S5).

### SCUB is heterogeneous along exons

**FIGURE 3:**
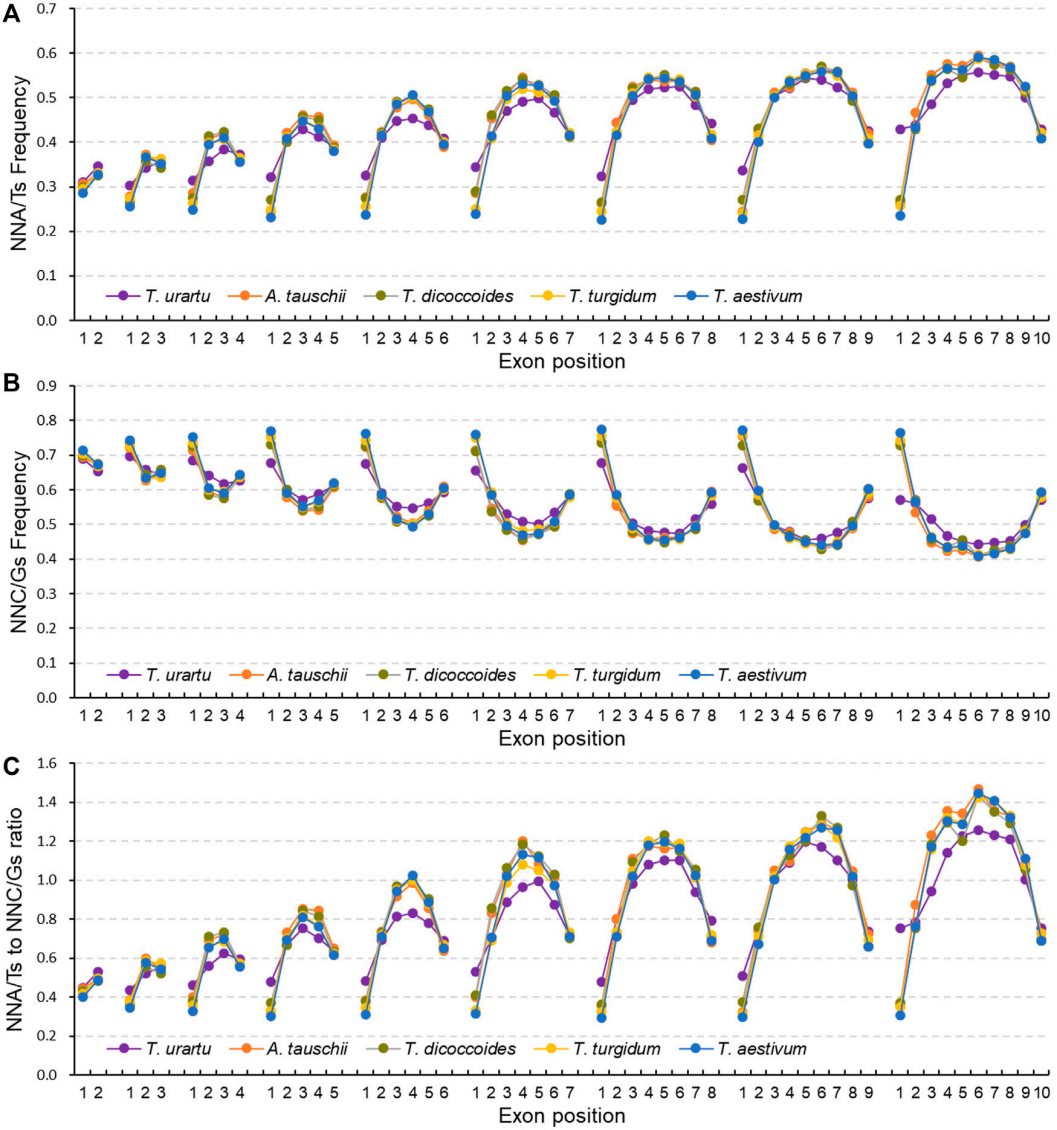
Heterogeneity of SCUB as a function of exon position in genes. Frequencies of A/T-ending SCs (NNA/Ts) **(A)** and C/G-ending SCs (NNC/Gs) **(B)** as a function of exon position in genes with one to nine introns. **(C)** Ratios between A/T-ending SCs and C/G-ending SCs (NNA/Ts and NNC/Gs) as a function of exon position in genes with one to nine introns. N denotes any base. The difference between hexaploid wheat and its progenitors was calculated by Chi square (χ^2^) test, and the results are presented in Supplementary Table S6.

Given the association between SCUB frequency and exon number, we further analyzed SCUB heterogeneity as a function of exon position along genes. In genes with two to ten exons, the frequencies of NNA, NNT and NNA/T in the first exon were lower than those seen in the last exon, while the frequencies of NNC, NNG and NNC/G showed the opposite pattern, with the exception of NNC frequencies in genes with exactly two exons (Figures 3A,B; Supplementary Figure S6, S7), resulting in lower NNA/T to NNC/G ratios in the first exon relative to the last exon (Figure 3C). Except for genes with ten exons in *T. urartu*, the frequencies of NNA, NNT, NNC, and NNG, as well as the ratios between NNA/T and NNC/G frequencies, were comparable across the first exons (CV = 0.011–0.087) (Supplementary Figure S6). The frequencies of NNA, NNT and NNA/T in the last exons gradually rose with the increase in exon number for genes with two to seven exons and were similar in genes with seven to ten exons, while the frequencies of NNC, NNG and NNC/G showed the opposite pattern (Figures 3A,B; Supplementary Figure S6, S7; Supplementary Table S6), such that the ratios between NNA/T and NNC/G frequencies gradually increased in genes with two to seven exons and then remained constant in genes with seven to ten exons (Figure 3C). In genes with three to ten exons, internal exons showed higher frequencies of NNA, NNT and NNA/T but lower frequencies of NNC, NNG and NNC/G when compared to terminal exons (Figure 3; Supplementary Figure S6, S7). Among internal exons, middle exons had the highest NNA, NNT and NNA/T frequencies but the lowest NNC, NNG and NNC/G frequencies, leading to convex curves (”∩“) for NNA, NNT and NNA/T frequencies, concave curves (”∪“) for NNC, NNG and NNC/G frequencies, and convex curves for the ratios between NNA/T and NNC/G frequencies. Moreover, the increase seen in NNA, NNT and NNA/T frequencies and concurrent decrease in NNC, NNG and NNC/G frequencies along internal exons appeared to correlate with both exon number and position (Supplementary Figure S6, S7). In genes with two to five exons, NNA/T frequencies were lower than those for NNC/G, with comparable frequencies for NNGs and NNTs in the middle exon positions for genes with five exons. In genes more than six exons, the frequencies of NNA, NNT and NNA/T were higher than those for NNC, NNG and NNC/G. For internal exons, the change in NNC and NNT frequencies was more drastic for NNG or NNA frequencies as exon number increased, with differences between NNA and NNT frequencies becoming larger while those for NNCs and NNGs decreased. Furthermore, in either tetraploid and hexaploid wheat, the NNC/G to NNA/T ratios from the first to last exons of genes with two to ten exons were similar among the subgenomes (Supplementary Figure S8).

In terminal exons, hexaploid wheat had the lowest NNA and NNT frequencies, the A subgenome diploid progenitor *T. urartu* the highest, and the tetraploid progenitors *T. dicoccoides* and *T. turgidum* intermediate frequencies; the difference in NNC and NNG frequencies among these species showed an opposite pattern (Figure 3). By contrast, NNA and NNT frequencies for internal exons were higher in hexaploid wheat and tetraploid *T. dicoccoides* and *T. turgidum* when compared to the diploid *T. urartu*, while NNC and NNG frequencies were lower; they were also comparable between hexaploid wheat and its tetraploid progenitors. In comparison to hexaploid wheat, the D subgenome diploid progenitor *A. tauschii* exhibited higher NNA and NNT frequencies but lower NNC and NNG frequencies in terminal exons, but similar frequencies for internal exons. For A subgenome, the NNC/G to NNA/T ratios from the first to last exon in genes with two to ten exons showed different between A subgenome progenitor *T. urartu* and polyploid wheat, while they were similar among tetraploid and hexaploid species (Supplementary Figure S9A). For B or D subgenome, the ratios were comparable among hexaploid and the progenitors (Supplementary Figure S9B).

### DNA methylation is involved in SCUB changes

**FIGURE 4:**
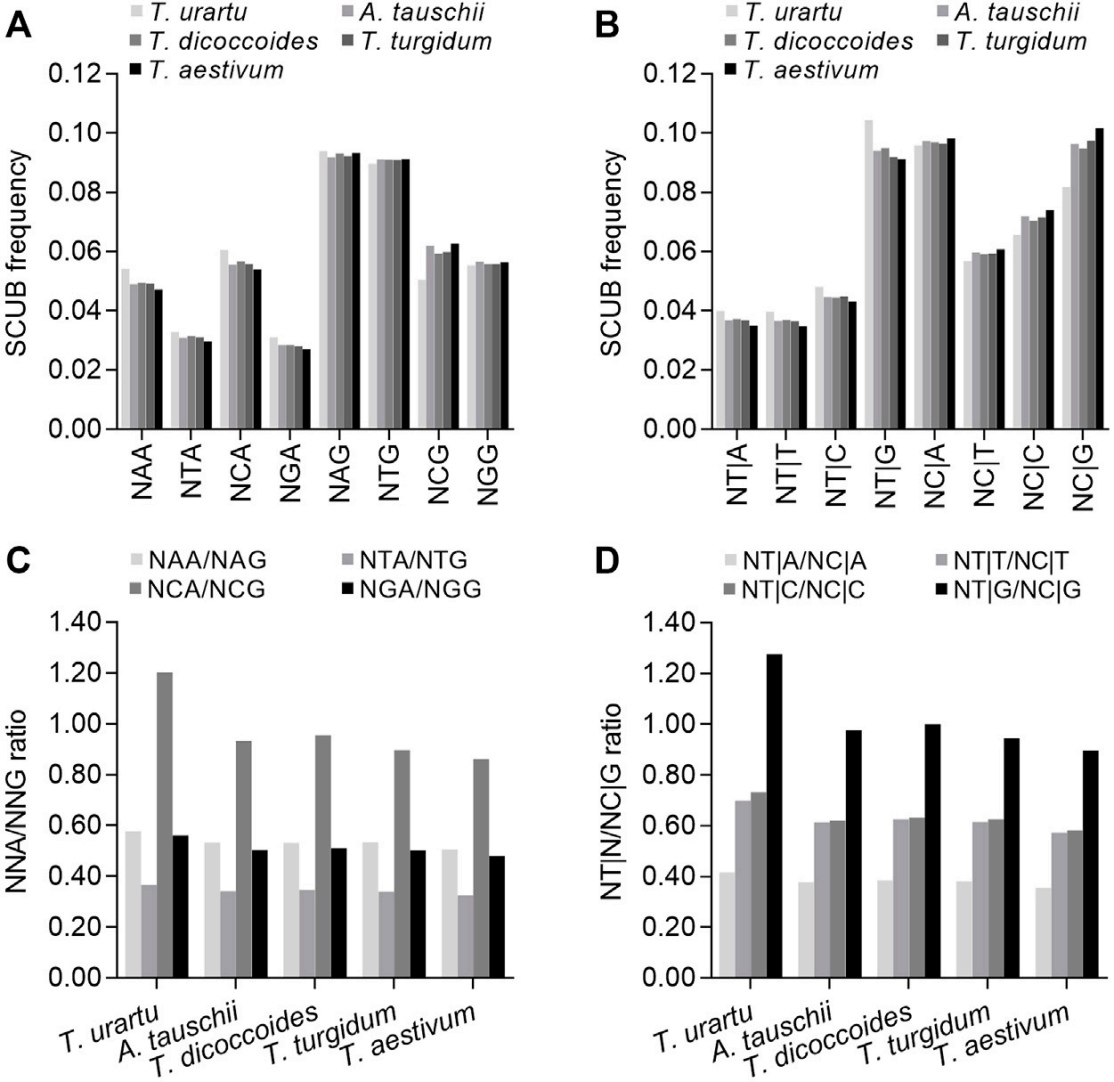
Association between SCUB and DNA methylation-driven conversion of cytosines to thymines.**(A)** SCUB frequencies of NNA and NNG indicating the effect of the second nucleotide position of codons on the conversion of C to T at the third position on the antisense strand. **(B)** SCUB frequencies of NT|N and NC|N indicating the effect of the first nucleotide position of the next codon on the conversion of C to T at the third position of the previous codon on the sense strand. **(C)** Ratios between NNA and NNG codon frequencies. **(D)** Ratios between NT|N and NC|N triplets. NNA and NNG: SCs with A and G as the final bases and any base at the second position; N denotes any base. NT|N and NC|N: SCs with C and T as the final base of the previous codon and any base at the first position of the next codon. The difference between hexaploid wheat and its progenitors was calculated by Chi square (χ2) test, and the results are presented in Supplementary Table S7.

The alteration of DNA methylation patterns is a typical genetic event following polyploidization, and methylation in the CpG sequence context may drive SCUB in the nuclear genome because methylated cytosine (5^m^C) is readily converted into thymine (Nabel et al., 2012). To investigate this possibility, we attempted to determine the influence on SC frequency of the nucleotide present in the second position of NNAs or NNGs (conversion of C to T on the antisense strand causing the conversion of G to A on the sense strand) and the nucleotide present in the first position of the downstream codon (NT|N and NC|N) (conversion of C to T on the sense strand). We first looked at codons ending with A or G: in *Triticum/Aegilops* spp. Genes, the frequencies of NAA, NCA, NGA and NTA were lower than those for NAG, NCG, NGG or NTG (*p* = 0.044 in *T. urartu* and *p* = 0.010–0.017 in other species) (Figure 4A). NCA frequencies were higher than NAA, NGA and NTA frequencies, while NCG frequencies were lower than NAG and NTG frequencies and comparable to those for NGGs. Thus, the ratios between NCA and NCG frequencies, indicative of methylation-mediated conversion of C to T on the antisense strand, were higher than the ratios between NAA/NAG, NGA/NGG or NTA/NTG frequencies (Figure 4C; Supplementary Table S7). The frequencies of NT|G triplets were higher than those for NT|A, NT|C or NT|T, while NC|G triplet frequencies were similar to those for NC|A triplets but higher than for NC|C or NC|G triplets, resulting in drastically higher NT|G/NC|G ratios, indicating methylation-mediated conversion of C to T on the sense strand relative to NT|A/NC|A, NT|C/NC|G and NT|T/NC|T ratios (Figures 4B,D; Supplementary Table S7). This result indicated that compared to triplets with A, G or T at the second position and A, C or T at the first nucleotide of the next codon, C at the second position and G at the first nucleotide of the next codon had a stronger effect on decreasing the bias of G and C appearing at the third position, suggesting the potential contribution of methylation-mediated conversion to SCUB. The SCUB based on the subgenome exhibited the similar profiles (Supplementary Figure S10A,B). The SCUB based on DNA methylation had similar patterns among the subgenomes in both tetraploid and hexaploid wheat (Supplementary Figure S10). Both NCA/NCG and NT|G/NC|G ratios of B subgenome were substantially higher than those of D subgenome among subgenomes in tetraploid and hexaploid wheat, but the ratios between A and D subgenomes were comparable.

NAA, NCA, NGA and NTA frequencies were the highest in diploid *T. urartu*, intermediate in tetraploid *T. dicoccoides* and *T. turgidum*, and the lowest in hexaploid wheat; NCG frequencies exhibited an opposite profile, while the difference in NAG, NGG or NTG frequencies among *Triticum/Aegilops* spp. was not as obvious as that in NCG frequencies (CV: 0.007–0.009 vs*.* 0.083; *p* value: 5.42 × 10^−72^–1.20 × 10^−42^ vs*.* 0, χ^2^ test). The ratios between NAA/NAG, NCA/NAG, NGA/NGG and NTA/NTG were also different among *Triticum/Aegilops* spp. In particular, the differences in the NCA/NCG ratios were more pronounced than those in the NAA/NAG, NGA/NGG and NTA/NTG ratios (CV: 0.139 vs*.* 0.043–0.059). Similarly, NT|A, NT|T, NT|C and NT|G triplet frequencies were the highest in diploid *T. urartu*, intermediate in tetraploid *T. dicoccoides* and *T. turgidum*, and the lowest in hexaploid wheat; NC|A, NC|C, NC|G and NC|T triplet frequencies followed the opposite pattern. We observed clear differences for the ratios between NT|G and NC|G, NT|A and NC|A, NT|C and NT|G, or NT|C and NT|G across *Triticum/Aegilops* spp., and differences in the ratios between NT|G and NC|G frequencies were more remarkable than for those in other three ratios (CV: 0.146 vs*.* 0.057–0.087). The predominant difference in the NCA/NCG and NT|G/NC|G ratios was consistent with the occurrence of methylation-mediated conversion. Furthermore, there had the significant difference in DNA associated SCUB in the same subgenome among hexaploid wheat and its progenitors (Supplementary Figure S10). Among NNA/NNG ratios, the NCA/NCG ratios had the most significant difference in A, B or D subgenomes, and they became smaller along with the rise of genome ploidy, as was also found when the NT|G/NC|G ratios were compared.

**FIGURE 5:**
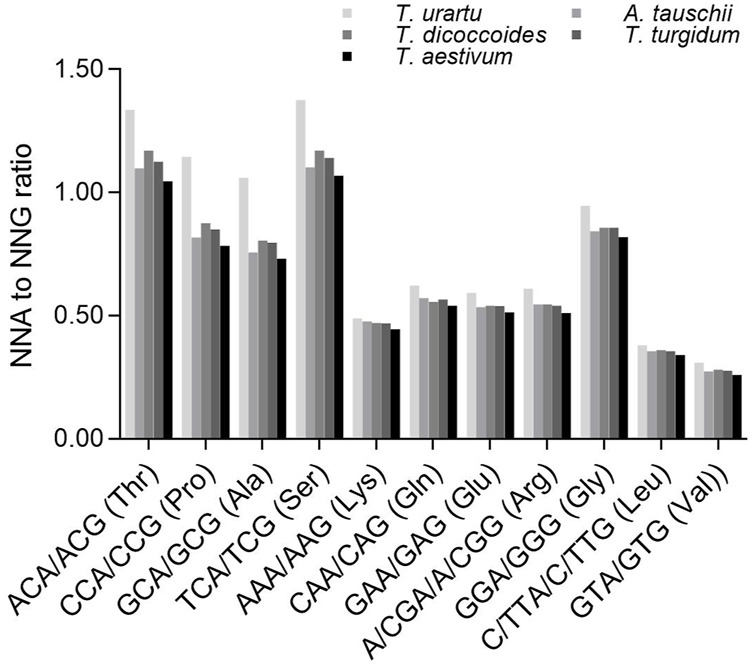
Ratios between A-ending SCs and G-ending SCs specifying various amino acids. The statistical comparison was conducted by Chi square (χ^2^) test, and the results are presented in Supplementary Table S8. The difference between the ratios for Ala, Pro, Ser, Thr and those for Arg, Glu, Gly, Leu, Lys, Val in a species was calculated with a two-sample Student’s *t*-test (*p* < 0.05).

We next analyzed a subset of C- and G-ending SC pairs encoding the same specifying amino acids that share the same nucleotides in their first and second positions to identify any effect of the second nucleotide on the SC frequency (Figure 5). The ratios between NCA and NCG frequencies (specifying Ala, Pro, Ser and Thr) varied from 0.731 to 1.375, which were significantly higher than the ratios between N (A/G/T)A and N (A/G/T)G frequencies (specifying Arg, Gly, Leu and Val) (0.260–0.622 except for Gly [0.818–0.946]) (*p* = 0.0003–0.004, *t*-test). The ratios between NCA and NCG or N (A/G/T)A and N (A/G/T)G were the highest in diploid *T. urartu*, the lowest in hexaploid wheat, and intermediate in tetraploid progenitors, but the difference across *Triticum/Aegilops* spp. was higher for the ratios between NCA and NCG (CV = 0.096–0.161) than for the ratios between N (A/G/T)A and N (A/G/T)G (CV = 0.035–0.066) (Supplementary Table S8).

**FIGURE 6:**
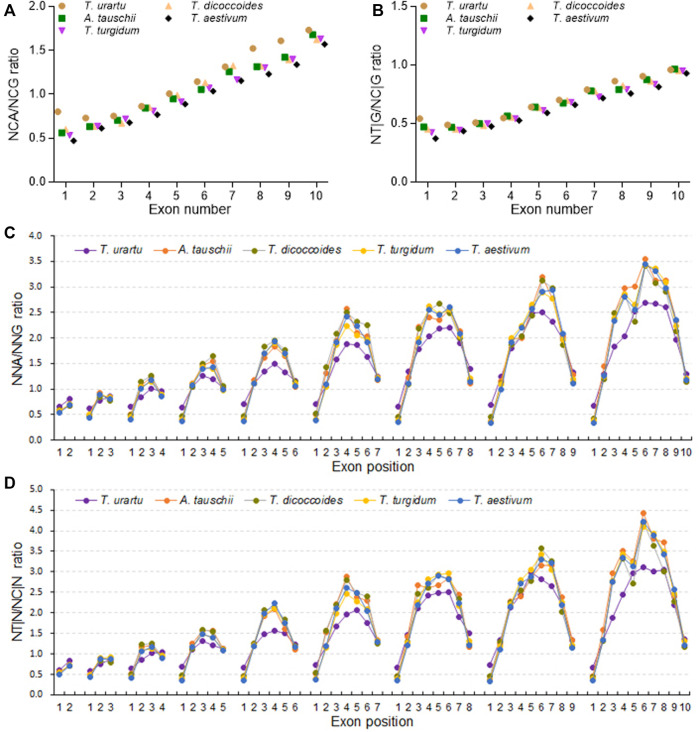
Association between DNA methylation and SCUB heterogeneity as a function of exon number and position. **(A)** Ratios between NCA and NCG codons in genes with up to nine introns. **(B)** Ratios between NT|G and NC|G triplets in genes with up to nine introns. **(C)** Ratios between NCA and NCG codons as a function of exon position in genes with one to nine introns. **(D)** Ratios between NT|N and NC|N triplets as a function of exon position in genes with one to nine introns. The difference was calculated by Chi square (χ^2^) test, and the results are presented in Supplementary Table S9.

The ratios between NAA and NAG, NCA and NCG, NGA and NGG, or NGA and NGG frequencies, as well as between NT|A and NC|A, NT|C and NC|C, NT|G and NC|G, or NT|T and NC|T triplet frequencies, all rose with increasing exon number (Figures 6A,B; Supplementary Figure S11). Among them, the increase in NCA/NCG ratios was sharper than those of the other three NNA/NNG ratios and held true when comparing the NT|G/NC|G ratios to the other NT|N/NC|N ratios (Supplementary Figure S11), indicating that DNA methylation-associated SCUB is more prominent in genes with more exons. The NCA/NCG and NT|G/NC|G ratios were the highest in diploid *T. urartu*, intermediate in tetraploid progenitors, and the lowest in hexaploid wheat (Supplementary Table S9). The ratios were also higher in the D subgenome diploid progenitor *A. tauschii* than in hexaploid wheat. The difference among *Triticum/Aegilops* spp. Appeared to be more significant in genes with two exons than in genes with more exons (CV: 0.212 and 0.217 vs*.* < 0.085). At subgenome level, the ratios of both NNC/NNG and NT|G/NC|G based on exon number were comparable among the subgenomes in either tetraploid and hexaploid wheat (Supplementary Figure S12). On the other hand, for A, B or D subgenome, the ratios of NCA/NCG in genes with one to ten exons were obviously different among hexaploid wheat, tetraploid wheat and diploid progenitors (Supplementary Figure S13). Among them, diploid progenitors had the highest ratios, hexaploid had the lowest ratios, and wild tetraploid wheat had higher ratios than domestic tetraploid wheat. However, the ratios of the other three NNA/NNG combinations based on exon number were similar among hexaploid wheat and its progenitors. The NT|N/NC|N ratios showed the same profiles, where the NT|G/NC|G ratios significantly decreased following the rise of genome polyploidy, but the others almost kept constant.

The ratios of NAA/NAG, NCA/NCG, NGA/NGG and NTA/NTG frequencies were almost constant for the first exons of genes with two to ten exons (Figure 6; Supplementary Figure S14). The ratios of NAA/NAG, NGA/NGG and NTA/NTG frequencies for the last exons were also comparable to those for the first exons, although the NCA/NCG ratios for the last exons gradually increased with higher exon number. The ratios of NAA/NAG, NCA/NCG, NGA/NGG and NTA/NTG frequencies for internal exons were higher than those of terminal exons, and the ratios for middle exons were the highest, resulting in convex curves (“∩”). The NCA/NCG ratios for internal exons were drastically higher than those for NAA/NAG, NGA/NGG and NTA/NTG (Supplementary Figure S14). The ratios between NT|A and NC|A, NT|C and NC|C, NT|G and NC|G, or NT|T and NC|T triplet frequencies among exons showed similar convex profiles, and the ratios between NT|G and NC|G triplet frequencies for internal exons was much higher than other ratios (Supplementary Figure S15). The NCA/NCG and NT|G/NC|G ratios for terminal exons were the highest in diploid *T. urartu*, intermediate in tetraploid *T. dicoccoides* and *T. turgidum*, and the lowest in hexaploid wheat, and the difference in the first exons among *Triticum/Aegilops* spp. increased with higher exon number. The ratios for internal exons exhibited an opposite profile, with ratios in diploid *T. urartu* being lower than in tetraploid or hexaploid species. In tetraploid and hexaploid wheat, the ratios based on exon position at subgenome level showed the same profiles, with significantly higher NCA/NCG and NT|G/NC|G ratios than the other NNA/NNG and NT|A/NC|N ratios in middle exons, but all the ratios among subgenomes were comparable (Supplementary Figure S17). For A subgenome, the ratios of NCA/NCG and NT|G/NC|G from the first to last exons in genes with two to ten exons were comparable among tetraploid and hexaploid wheat, but they were different from those of diploid *T. urartu* (Supplementary Figure S17). For B and D subgenomes, the ratios of NCA/NCG and NT|G/NC|G were similar among the species. The ratios of the other NNA/NNG and NT|N/NC|N ratios were almost the same among hexaploid wheat and its progenitors in A, B and D subgenomes.

C- and G-ending SC pairs encoding the same specifying amino acids that share the same nucleotides in their first and second positions also confirmed the effect of DNA methylation on SCUB (Supplementary Figure S18, S19). NCA/NCG (specifying Ala, Pro, Ser and Thr) ratios were higher than those between N (A/G/T)A and N (A/G/T)G (specifying Arg, Gly, Leu and Val), and this difference increased with higher exon number (Supplementary Figure S18). Moreover, NCA/NCG ratios of Ala, Pro, Ser and Thr among exons exhibited sharp convex curves, but the ratios between N (A/G/T)A and N (A/G/T)G (Arg, Gly, Leu and Val) formed moderate convex curves (Supplementary Figure S19), which was consistent with those of NCA/NCG ratios as well as NAA/NAG, NGA/NGG and NTA/NTG ratios among exons (Supplementary Figure S15).

### SCUB mirrors the effects of polyploidization

We performed a clustering analysis of *Triticum/Aegilops* spp. based on SCUB frequencies (Supplementary Figure S20A). The A subgenome diploid progenitor *T. urartu* formed a separate clade away from all other species. In the other clade, the D subgenome progenitor *A. tauschii* and tetraploid progenitors defined a sub-clade that clustered away from the hexaploid wheat. This analysis was supported by the outcome of principal component analysis (PCA) (Supplementary Figure S20B,C). The first and second principal components (PC1 and PC2) distinguished the A subgenome progenitor, the D and AB subgenome progenitors, and hexaploid wheat, which aligned as a function of ploidy. A subgenome progenitor *T. urartu* was associated with the smallest factor score coefficients (FSC) and was well separated from the remaining *Triticum/Aegilops* spp. The D subgenome progenitor was separated from the AB subgenome progenitors and hexaploid wheat along PC3 (Supplementary Figure S20C). Together with the phylogenic data and PCA, SCUB can therefore reflect the differences between polyploids and their ancestors.

## Discussion

SCUB varies across the nuclear genomes of land plants, with a bias toward either NNA/T or NNC/G. Hexaploid wheat and its progenitors all preferred to use NNC/G (Figure 1). Polyploidization is one of the most important evolutionary events in plants and can increase genetic diversity and introduce new genetic combinations. Polyploidization leads to global genetic variation due to the immediate redundancy between homeologs, including nucleotide substitutions (Jiao et al., 2011), which may alter SCUB. Here, we discovered that following an increase in genome ploidy, codons specifying the same amino acid preferred those SCs ending with C or G, resulting in their higher frequencies (Figure 1). The difference may be associated with their different genome nature and gene abundance, because the difference in SCUB among wheat and its diploid and tetraploid progenitors based on a subgenome was similar as the difference based on the whole genome (Supplementary Figure S3). This observation demonstrates that polyploidization affects SCUB and promotes the preference toward NNC/G in wheat. On the other hand, the difference in SCUB frequencies was also observed based on the orthologous genes (Supplementary Table S10), which indicates that increased codon usage bias is, at least partially, due to polyploidy, and arose in connection with the formation of hexaploid wheat. Moreover, CAI was also different among wheat and its progenitors (Supplementary Table S4), implying that differential annotation of genes with greater or lesser codon usage bias in the reference genomes of these species.

Intron evolution is a major event in eukaryotic genomes (Fawcett et al., 2012), and causes nuclear substitutions in exon sequences, which commonly prefers lower GC content (Singh et al., 2005). In wheat and its progenitors, the bias toward NNA/Ts became more pronounced with higher intron numbers (Figure 2). Genes with more introns may be subjected to stronger selective pressure, such that they will tend to favor the retention of NNA/T (Bernardi 2000; Xing and Lee 2006). However, although genomic variation following polyploidization is under selection (Qiao et al., 2019), the frequencies of NNA/T decreased with the rise in genome polyploidy for genes with up to nine introns (Figure 2). This result suggests that selective pressure appear to have a neutral effect on the relationship between intron number and SCUB. The difference in the frequencies of NNA/T between wheat and its progenitors diminished in genes harboring more introns, suggesting the presence of a minor effect from selective pressure on intron number following wheat polyploidization.

Intron evolution is a type of sequence InDel polymorphism and may induce nucleotide substitutions in and around the flanking exons (Tian et al., 2008; Zhang et al., 2008). Higher bias for NNA/T in internal exons compared to terminal exons was apparent in the genes of hexaploid wheat and its progenitors (Figure 3), and the bias in internal exons was more pronounced in genes with more introns, consistent with the increase in the bias for NNA/T as a function of greater intron number (Figure 2). In line with the constant bias for NNA/T in terminal exons (Figure 2), our finding further confirms that internal introns are a major contributor to the effect of introns on SCUB. One possible cause may be that evolution gain and loss largely occur in the middle part of genes, which induces genetic and epigenetic variation of adjacent sequences and promotes the bias to NNA/T. Moreover, whether the higher bias to NNA/T in middle exons is associated with the function of middle exons needs to be further investigated. In line with the associated between SCUB and intron number/position during plant evolution (Qin et al., 2013) and the formation of polyploid wheat, it could be concluded that the effect of SCUB is a common genetic event in the genomic variation.

Besides genetic variation, epigenetic variation such as DNA methylation often accompanies polyploidization of natural allopolyploids (Comai 2000; Shaked et al., 2001; Kashkush et al., 2002; Kashkush et al., 2003; Scarrow et al., 2020) or newly synthesized allohexaploid wheat (Shaked et al., 2001). Given that methylated cytosine can be converted to thymine (Ossowski et al., 2010), DNA methylation is a source of polymorphisms (Laird 2010). Here, DNA methylation was closely associated with SCUB as well as the effect of intron number and exon position on SCUB in wheat and its progenitors, as indicated by the comparison of the ratios of NCA/NCG and NXA/NXG (X = A, G and T) as well as NT|G/NC|G and NT|X/NC|X (X = A, C and T) (Figures 4–6). This result confirms the contribution of DNA methylation to SCUB in plants. Furthermore, the ratios of the frequencies of NCA/NCG and NT|G/NC|G decreased following the increase in genome ploidy (Figures 4, 5) and coincided with the observed differences in SCUB between hexaploid wheat and its progenitors (Figure 1). However, we obtained the result opposite to our expectations that hexaploid wheat should have higher NCA/NCG and NT|G/NC|G ratios than its progenitors, because DNA methylation typically increases the conversion rate of C to T. This finding implies that although DNA methylation promotes SCUB, it may not be the main driving force for the shift in SCUB following polyploidization.

On the contrary, DNA methylation appears to be dynamic and reversible upon a change in genome ploidy (Yuan et al., 2020). In extracted tetraploid wheat derived from natural hexaploid wheat, DNA methylation levels decreases, while in resynthesized hexaploid wheat derived from extracted tetraploid wheat, DNA methylation levels increases (Yuan et al., 2020). The frequencies of NNC and NNG became stronger following the rise of genome ploidy (Supplementary Figure S3), showing that during the formation of wheat polyploidies, SCUB of all subgenomes prefers to the bias for C and G-ending codons. It should be noted that the SCUB frequencies of A subgenome progenitor *T. urartu* and D subgenome progenitor *A. tauschii* were obviously distinct, but the SCUB frequencies of A and D subgenomes were comparable in hexaploid wheat. In line with the similar SCUB frequencies among the subgenomes in both tetraploid and hexaploid wheat, it could be implied that genomic variation (nucleotide substitution) in the formation of wheat polyploidies may be not a random genetic event, which partially leads to homogeneity of SCUB of subgenomes from different progenitors. Moreover, SCUB difference indicated by the ratios of NCA/NCG and NT|G/NC|G but not the ratios of the other NNA/NNG and NT|N/NC|N at subgenome level (Supplementary Figure S10–S13) further confirms that SCUB alteration may contributes to DNA methylation in polyploid wheat. Consistently, the methylation levels of A, B and D subgenomes are comparable with each other (Gardiner et al., 2015; Yuan et al., 2020), and the gain of methylation in three subgenomes are all higher than the loss of methylation (Gardiner et al., 2015). Thus, we speculate that the decrease in DNA methylation-mediated SCUB shift may promote the conversion of T to C, so as to produce methylation sites to modulate global DNA methylation levels.

In polyploidies, dosage effects are caused by the increase in the number of chromosome copies, genomic rearrangement and InDels result in changes in gene expression (Shi et al., 2020). Epigenetic variation such as DNA methylation governs the balance of gene expression (Mutti et al., 2017) to achieve subgenome expression asymmetry (Yang et al., 2021). Moreover, given that synonymous mutations are mostly strongly non-neutral (Shen et al., 2022) and SCs affect transcription efficiency, mRNA stability, translational efficiency and accuracy (Zhang *et al.*; Marais et al., 2001; Warnecke and Hurst 2007; Tuller et al., 2010; Presnyak et al., 2015), a shift in SCUB may be detrimental to the phenotype of polyploidies. Thus, the substitution between SCs can also be used for mining genes and excellent allelic variation governing agricultural traits of wheat and other crops.

Both phylogenetic analysis and PCA illustrated the heterogeneity of SCUB patterns between hexaploid wheat and its progenitors (Supplementary Figure S20). Especially, the distribution of the A subgenome progenitor *T. urartu*, tetraploid wheat and hexaploid wheat along the first two PCs mirrored the formation of hexaploid wheat (Supplementary Figure S20B) and confirmed the shift in SCUB as an evolutionary event following polyploidization. SCUB in *T. urartu* differed markedly from that seen in the AB subgenome tetraploid progenitor and ABD hexaploid wheat. Given that SCUB reflects a balance between mutation, genetic drift and natural selection (Akashi and Eyre-Walker 1998; Akashi 2001; Guo and Yuan 2009; Wang et al., 2014), and the B subgenome progenitor and its genome sequence is unknown, the distinctive difference between SCUB in *T. urartu* and that in tetraploid/hexaploid wheat may be due to a distinct SCUB pattern in the B subgenome progenitor. Moreover, SCUB may have experienced different selection pressures over the course of the two rounds of allopolyploidization involved in the formation of hexaploid wheat. During the formation of polyploidies, the diploidization of the genomes is achieved *via* a set of genetic and epigenetic variation such as chromosome rearrangement, large sequence elimination, insertion and deletion, transposon activation, DNA methylation alteration (Feldman and Levy 2012), the association between SCUB and genetic/epigenetic variation of polyploidies must be complicated. In summary, our results suggest that there have two effects on SCUB during the formation of polyploid wheat: the major effect of the bias to C/G-ending codons, and the minor effect of the bias to A/T-ending codons *via* intron evolution.

## Experimental procedures

### Genome sequences and codon counts

The genomes from hexaploid bread wheat (*Triticum aestivum*, AABBDD), its wild tetraploid progenitor *T. dicoccoides* (AABB) and domesticated tetraploid progenitor *T. turgidum* (AABB), the A subgenome progenitor *T. urartu* (AA) and the D subgenome progenitor *Aegilops tauschii* (DD) were used for analysis. Their genome sequences were downloaded from the EnsemblPlants database (http://plants.ensembl.org/info/data/ftp/index.html). Coding sequences were extracted according to the GFF3 gene annotation files also downloaded from the EnsemblPlants database using the TBtools (Chen et al., 2020). For genes with more than one gene model, the first transcript was used for analysis. Extracted coding sequences whose lengths were not multiples of 3, those that contained N, and those with a start codon different from ATG or stop codons distinct from TAA, TAG or TGA were excluded from this analysis. Codons interrupted by an intron between the first and the second nucleotides were treated as belonging to the downstream exon, while those interrupted between the second and the third nucleotides were deemed to belong to the upstream exon.

### Calculation of SCUB indices

All extracted coding sequences for one of the genomes mentioned above were combined into one FASTA file, which was used to calculate relative synonymous codon usage (RSCU), codon adaptation index (CAI) and other SCUB indices with CodonW 1.4.2 software (http://codonw.sourceforge.net/). CAI indicates that what extent codon usage of a gene is adapted toward the codon usage of highly expressed genes in a genome (Sharp and Li, 1987). RSCU is defined as the observed frequency of a given codon divided by its expected frequency in the absence of usage bias (which is the average frequency of all codons for that amino acid), and could directly reflect the bias of codon use (Sharp et al., 1986).

### Calculation of SCUB frequencies

We also calculated SCUB frequencies as a representation of the bias in SCs. The frequency for all 61 codons (omitting the three stop codons) was calculated using the ratio between the number of occurrences for each codon and the number of all codons from the extracted coding sequences of the whole genome. In addition, the 59 SCs corresponding to 18 of the 20 amino acids were used to calculate SCUB frequency. The three stop codons TAA, TAG and TGA were excluded from this analysis; the start codon ATG for methionine and the TGG codon for tryptophan were also excluded, as they do not have SCs. The number of SCs in all coding sequences across one species was calculated from the number of all non-unique and non-stop codons. The frequency of a given SC was calculated as the ratio between the number of this given SC to the number of 59 SCs in a species. The SCUB frequency for each amino acid specified by SCs was calculated as the ratio between the number of C/G-ending SCs specifying a given amino acid to the number of A/T-ending SCs specifying the amino acid. The total SCUB frequency was calculated as the ratio between the number of all SCs having A, T, C or G at their third position (abbreviated NNA, NNT, NNC or NNG, respectively) and the number of all codons across all coding sequences, omitting the start codon, stop codons and TGG.

SCs for a given amino acid differ at the third position, which also experiences lower selection pressure. CpG-type methylation would therefore convert NCG codons to NCA (if the cytosine on the antisense strand is methylated) and NC|G triplets to NT|G (when the sense cytosine is methylated), which leads to a bias for A/T-ending codons. Thus, the ratios between the numbers of NXA and NXG codons (X = A, T, C, or G) can indicate the effect of the second nucleotide on the conversion from G and C to A and T at the third position, and the ratios between the numbers of NG|X and NC|X triplets (X = A, T, C, or G) can indicate the effect of the first nucleotide from the next codon on the conversion from G and C to A and T at the third position. The difference between the ratios of NCA/NCG and NAA/NAG, NGA/NGG, NTA/NTG as well as the difference between the ratios of NT|G/NC|G and NT|A/NC|A, NT|C/NC|C and NT|T/NC|T were thus compared to evaluate the association between DNA methylation and SCUB.

### Cluster analysis and PCA

Cluster analysis using SC frequencies and RSCU values from all 59 SCs was conducted with the average linkage method in Minitab 17 statistical software (Minitab Inc.). The dendrogram was generated on the basis of similarity. SCUB frequencies and RSCU values for the 59 SCs were also subject to PCA in JMP 13 software (SAS Inc.) with default parameters. The factor score coefficients given by the first three PCs were used to generate scatter plots.

### Extraction of CDS of orthologous genes

The genomes among wheat and its diploid and tetraploid progenitors were subject to collinearity analysis to get the orthologous genes among this species (Chen et al., 2020). Among them, the genome of *T. urartu* and the A subgenomes of hexaploid wheat and tetraploid progenitors were analyzed together to get orthologous genes of A subgenome, the B subgenomes of hexaploid wheat and tetraploid progenitors together to get orthologous genes of B subgenome, the genome of *A. tauschii* and the D subgenome of hexaploid wheat together to get orthologous genes of D subgenome. The orthologous genes were confirmed by local BLAST. The CDS of orthologous genes were extracted, filtered and used for SCUB frequency as mentioned above.

### Statistical analysis

The difference between SCUB frequencies for NNA, NNT, NNC and NNG for a given species was calculated using the Chi square (χ^2^) test, using the numbers of NNA, NNT, NNC, and NNG for calculation. The difference between SCUB frequencies for NNA/T and NNC/G for a given species was calculated using the Chi square (χ^2^) test, using the numbers of NNA/T and NNC/G for calculation. The difference in SCUB frequency for NNA, NNT, NNC or NNG as well as NNA/T or NNC/G between hexaploid wheat and its progenitors was calculated using the Chi square (χ^2^) test of the cross-table analysis, using the numbers of NNA, NNT, NNC, NNG, NNA/T or NNC/G and all SCs for calculation; the difference between two species was calculated with Chi square partitioning. The difference in SCUB frequency related to the third nucleotide position in regard to DNA methylation was analyzed with the Chi square (χ^2^) test of the cross-table analysis. For example, the difference between the NCA/NCG ratio and the NXA/NXG ratio (X = A, G or T) was calculated from the numbers of NCA, NCG, NXA and NXG three-nucleotide triplets; the difference between the NC|G/NG|G ratio (with | indicating the separation between consecutive codons) and the NC|X/NG|X ratio (X = A, C, or T) was calculated from the numbers of NC|G, NG|G, NC|X and NG|X three-nucleotide triplets. The difference between NXC and NXG SCs for a given amino acid specified by G/C-ending SCs (Ala, Pro, Ser, Thr, Arg, Gly, Leu and Val) was calculated with the Chi square (χ^2^) test, with the numbers of NXC and NXG three-nucleotide triplets used for calculation. The difference between the NCG/NCA ratios for Ala, Pro, Ser, and Thr and the N (G/T)G/N (G/T)A ratios for Arg, Gly, Leu or Val was calculated with the *t*-test. The difference in SCUB frequencies between genes with different intron numbers as well as between exons was calculated by two-sample *t*-tests, where the ratios between NNC/G and NNA/T in genes with different intron numbers as well as the ratios between NNC/G and NNA/T in different exons were used for comparison. The difference in SCUB frequency between genes with different intron numbers and between exons in *Triticum/Aegilops* spp. was calculated by two-sample *t*-tests, and the NNC/G to NNA/T ratios were used for analysis. The fluctuation of SCUB frequencies was assessed by calculating the CV, which is the ratio between the standard deviation and the mean. The correlation of SCUB frequencies for all 18 amino acids (omitting the amino acids with single codons, Met and Trp) between two species were analyzed by Pearson’s correlation coefficient analysis. *p* values below 0.05 were considered significant.

## Data Availability

The datasets presented in this study can be found in online repositories. The names of the repository/repositories and accession number(s) can be found in the article/Supplementary Material.

## References

[B1] Adams K. (2007). Evolution of duplicate gene expression in polyploid and hybrid plants. J. Hered. 98, 136–141. 10.1093/jhered/esl061 17208934

[B2] Akashi H. Eyre-Walker A. (1998). Translational selection and molecular evolution. Curr. Opin. Genet. Dev. 8, 688–693. 10.1016/s0959-437x(98)80038-5 9914211

[B3] Akashi H. (2001). Gene expression and molecular evolution. Curr. Opin. Genet. Dev. 11, 660–666. 10.1016/s0959-437x(00)00250-1 11682310

[B4] Bernardi G. (2000). Isochores and the evolutionary genomics of vertebrates. Gene 241, 3–17. 10.1016/s0378-1119(99)00485-0 10607893

[B5] Bevan M. Uauy C. Wulff B. Zhou J. Krasileva K. Clark M. (2017). Genomic innovation for crop improvement. Nature 543, 346–354. 10.1038/nature22011 28300107

[B6] Bonen L. Vogel J. (2001). The ins and outs of group II introns. Trends Genet. 17, 322–331. 10.1016/s0168-9525(01)02324-1 11377794

[B7] Chen C. Chen H. Zhang Y. Thomas H. R. Frank M. H. He Y. (2020). TBtools: An integrative toolkit developed for interactive analyses of big biological data. Mol. Plant 13, 1194–1202. 10.1016/j.molp.2020.06.009 32585190

[B8] Chen J.-Q. Wu Y. Yang H. Bergelson J. Kreitman M. Tian D. (2009). Variation in the ratio of nucleotide substitution and indel rates across genomes in mammals and bacteria. Mol. Biol. Evol. 26, 1523–1531. 10.1093/molbev/msp063 19329651

[B9] Chen Y. Song W. Xie X. Wang Z. Guan P. Peng H. (2020). A collinearity-incorporating homology inference strategy for connecting emerging assemblies in the triticeae tribe as a pilot practice in the plant pangenomic era. Mol. Plant 13, 1694–1708. 10.1016/j.molp.2020.09.019 32979565

[B10] Chen Z. J. Ni Z. (2006). Mechanisms of genomic rearrangements and gene expression changes in plant polyploids. Bioessays 28, 240–252. 10.1002/bies.20374 16479580 PMC1986666

[B11] Cheng F. Wu J. Cai X. Liang J. Freeling M. Wang X. (2018). Gene retention, fractionation and subgenome differences in polyploid plants. Nat. Plants 4, 258–268. 10.1038/s41477-018-0136-7 29725103

[B12] Choi K. Weng M.-L. Ruhlman T. A. Jansen R. K. (2021). Extensive variation in nucleotide substitution rate and gene/intron loss in mitochondrial genomes of Pelargonium. Mol. Phylogenet. Evol. 155, 106986. 10.1016/j.ympev.2020.106986 33059063

[B13] Comai L. (2000). Genetic and epigenetic interactions in allopolyploid plants. Plant Mol. Biol. 43, 387–399. 10.1023/a:1006480722854 10999418

[B14] Coulombe-Huntington J. Majewski J. (2007). Characterization of intron loss events in mammals. Genome Res. 17, 23–32. 10.1101/gr.5703406 17108319 PMC1716263

[B15] Fawcett J. A. Rouzé P. Van de Peer Y. (2012). Higher intron loss rate in *Arabidopsis thaliana* than *A. lyrata* is consistent with stronger selection for a smaller genome. Mol. Biol. Evol. 29, 849–859. 10.1093/molbev/msr254 21998273

[B16] Feldman M. Levy A. A. (2012). Genome evolution due to allopolyploidization in wheat. Genetics 192, 763–774. 10.1534/genetics.112.146316 23135324 PMC3522158

[B17] Freeling M. Scanlon M. J. Fowler J. E. (2015). Fractionation and subfunctionalization following genome duplications: Mechanisms that drive gene content and their consequences. Curr. Opin. Genet. Dev. 35, 110–118. 10.1016/j.gde.2015.11.002 26657818

[B18] Gardiner L.-J. Quinton-Tulloch M. Olohan L. Price J. Hall N. Hall A. (2015). A genome-wide survey of DNA methylation in hexaploid wheat. Genome Biol. 16, 273. 10.1186/s13059-015-0838-3 26653535 PMC4674939

[B19] Giroux M. J. Clancy M. Baier J. Ingham L. McCarty D. Hannah L. C. (1994). De novo synthesis of an intron by the maize transposable element Dissociation. Proc. Natl. Acad. Sci. U. S. A. 91, 12150–12154. 10.1073/pnas.91.25.12150 7991598 PMC45394

[B20] Guo F. B. Yuan J. B. (2009). Codon usages of genes on chromosome, and surprisingly, genes in plasmid are primarily affected by strand-specific mutational biases in *Lawsonia intracellularis* . DNA Res. 16, 91–104. 10.1093/dnares/dsp001 19221094 PMC2671203

[B21] Hershberg R. Petrov D. A. (2008). Selection on codon bias. Annu. Rev. Genet. 42, 287–299. 10.1146/annurev.genet.42.110807.091442 18983258

[B22] Iñiguez L. P. Hernández G. (2017). The evolutionary relationship between alternative splicing and gene duplication. Front. Genet. 8, 14. 10.3389/fgene.2017.00014 28261262 PMC5306129

[B23] Jiao Y. Wickett J. N. Ayyampalayam S. Chanderbali A. S. Landherr L. Ralph P. E. (2011). Ancestral polyploidy in seed plants and angiosperms. Nature 473, 97–100. 10.1038/nature09916 21478875

[B24] Kashkush K. Feldman M. Levy A. A. (2002). Gene loss, silencing and activation in a newly synthesized wheat allotetraploid. Genetics 160, 1651–1659. 10.1093/genetics/160.4.1651 11973318 PMC1462064

[B25] Kashkush K. Feldman M. Levy A. A. (2003). Transcriptional activation of retrotransposons alters the expression of adjacent genes in wheat. Nat. Genet. 33, 102–106. 10.1038/ng1063 12483211

[B26] King J. Jukes T. (1969). Non-Darwinian evolution. Science 165, 788–798. 10.1126/science.164.3881.788 5767777

[B27] Knowles D. G. McLysaght A. (2006). High rate of recent intron gain and loss in simultaneously duplicated *arabidopsis* genes. Mol. Biol. Evol. 23, 1548–1557. 10.1093/molbev/msl017 16720694

[B28] Laird P. W. (2010). Principles and challenges of genome-wide DNA methylation analysis. Nat. Rev. Genet. 11, 191–203. 10.1038/nrg2732 20125086

[B29] Li N. Xu C. Zhang A. Lv R. Meng X. Lin X. (2019). DNA methylation repatterning accompanying hybridization, whole genome doubling and homoeolog exchange in nascent segmental rice allotetraploids, New Phytol. 223. 10.1111/nph.15820 30919978

[B30] Marais G. Mouchiroud D. Duret L. (2001). Does recombination improve selection on codon usage? Lessons from nematode and fly complete genomes. Proc. Natl. Acad. Sci. U. S. A. 98, 5688–5692. 10.1073/pnas.091427698 11320215 PMC33274

[B31] McClintock B. (1984). The significance of responses of the genome to challenge. Science 226, 792–801. 10.1126/science.15739260 15739260

[B32] Mourier T. Jeffares D. C. (2003). Eukaryotic intron loss. Science 300, 1393. 10.1126/science.1080559 12775832

[B33] Mutti J. S. Bhullar R. K. Gill K. S. (2017). Evolution of gene expression balance among homeologs of natural polyploids. G3 (Bethesda) 7, 1225–1237. 10.1534/g3.116.038711 28193629 PMC5386871

[B34] Nabel C. S. Manning S. A. Kohli R. M. (2012). The curious chemical Biology of cytosine: Deamination, methylation, and oxidation as modulators of genomic potential. ACS Chem. Biol. 7, 20–30. 10.1021/cb2002895 22004246 PMC3262930

[B35] Nei M. Gojobori T. (1986). Simple methods for estimating the numbers of synonymous and nonsynonymous nucleotide substitutions. Mol. Biol. Evol. 3, 418–426. 10.1093/oxfordjournals.molbev.a040410 3444411

[B36] Ossowski S. Schneeberger K. Lucas-Lledó J. I. Warthmann N. Clark R. M. Shaw R. G. (2010). The rate and molecular spectrum of spontaneous mutations in *Arabidopsis thaliana* . Science 327, 92–94. 10.1126/science.1180677 20044577 PMC3878865

[B38] Presnyak V. Alhusaini N. Chen Y. Martin S. Morris N. Kline N. (2015). Codon optimality is a major determinant of mRNA stability. Cell 160, 1111–1124. 10.1016/j.cell.2015.02.029 25768907 PMC4359748

[B39] Qiao X. Li Q. Yin H. Qi K. Li L. Wang R. (2019). Gene duplication and evolution in recurring polyploidization-diploidization cycles in plants. Genome Biol. 20, 38. 10.1186/s13059-019-1650-2 30791939 PMC6383267

[B40] Qin Z. Cai Z. Xia G. Wang M. (2013). Synonymous codon usage bias is correlative to intron number and shows disequilibrium among exons in plants. BMC Genomics 14, 56. 10.1186/1471-2164-14-56 23350908 PMC3576282

[B41] Rodríguez-Trelles F. Tarrío R. Ayala F. J. (2006). Origins and evolution of spliceosomal introns. Annu. Rev. Genet. 40, 47–76. 10.1146/annurev.genet.40.110405.090625 17094737

[B42] Salamini F. Ozkan H. Brandolini A. Schafer-Pregl R. Martin W. (2002). Genetics and geography of wild cereal domestication in the near east. Nat. Rev. Genet. 3, 429–441. 10.1038/nrg817 12042770

[B43] Scarrow M. Wang Y. Sun G. (2020). Molecular regulatory mechanisms underlying the adaptability of polyploid plants. Biol. Rev. Camb. Philos. Soc. 96, 394–407. 10.1111/brv.12661 33098261

[B44] Shaked H. Kashkush K. Ozkan H. Feldman M. Levy A. A. (2001). Sequence elimination and cytosine methylation are rapid and reproducible responses of the genome to wide hybridization and allopolyploidy in wheat. Plant Cell 13, 1749–1759. 10.1105/tpc.010083 11487690 PMC139131

[B45] Sharp P. M. Li W. H. (1987). The codon adaptation index-a measure of directional synonymous codon usage bias, and its potential applications. Nucleic Acids Res. 15, 1281–1295. 10.1093/nar/15.3.1281 3547335 PMC340524

[B46] Sharp P. M. Tuohy T. M. Mosurski K. R. (1986). Codon usage in yeast: Cluster analysis clearly differentiates highly and lowly expressed genes. Nucleic Acids Res. 14, 5125–5143. 10.1093/nar/14.13.5125 3526280 PMC311530

[B47] Sharpton T. J. Neafsey D. E. Galagan J. E. Taylor J. W. (2008). Mechanisms of intron gain and loss in *Cryptococcus* . Genome Biol. 9, R24. 10.1186/gb-2008-9-1-r24 18234113 PMC2395259

[B48] Shen X. Song S. Li C. Zhang J. (2022). Synonymous mutations in representative yeast genes are mostly strongly non-neutral. Nature 606, 725–731. 10.1038/s41586-022-04823-w 35676473 PMC9650438

[B49] Shi X. Chen C. Yang H. Hou J. Ji T. Cheng J. (2020). The gene balance hypothesis: Epigenetics and dosage effects in plants. Methods Mol. Biol. 2903, 161–171. 10.1007/978-1-0716-0179-2_12 32088896

[B50] Singh N. D. Arndt P. F. Petrov D. A. (2005). Genomic heterogeneity of background substitutional patterns in *Drosophila melanogaster* . Genetics 169, 709–722. 10.1534/genetics.104.032250 15520267 PMC1449091

[B51] Song Q. Chen Z. (2015). Epigenetic and developmental regulation in plant polyploids. Curr. Opin. Plant Biol. 24, 101–109. 10.1016/j.pbi.2015.02.007 25765928 PMC4395545

[B52] Stoltzfus A. (2004). Molecular evolution: Introns fall into place. Curr. Biol. 14, R351–R352. 10.1016/j.cub.2004.04.024 15120089

[B53] Tarrío R. Ayala F. J. Rodríguez-Trelles F. (2008). Alternative splicing: A missing piece in the puzzle of intron gain. Proc. Natl. Acad. Sci. U. S. A. 105, 7223–7228. 10.1073/pnas.0802941105 18463286 PMC2438231

[B54] Tian D. Wang Q. Zhang P. Araki H. Yang S. Kreitman M. (2008). Single-nucleotide mutation rate increases close to insertions/deletions in eukaryotes. Nature 455, 105–108. 10.1038/nature07175 18641631

[B55] Tuller T. Carmi A. Vestsigian K. Navon S. Dorfan Y. Zaborske J. (2010). An evolutionarily conserved mechanism for controlling the efficiency of protein translation. Cell 141, 344–354. 10.1016/j.cell.2010.03.031 20403328

[B56] Van de Peer Y. Fawcett J. A. Proost S. Sterck L. Vandepoele K. (2009). The flowering world: A tale of duplications. Trends Plant Sci. 14, 680–688. 10.1016/j.tplants.2009.09.001 19818673

[B57] Van de Peer Y. Mizrachi E. Marchal K. (2017). The evolutionary significance of polyploidy. Nat. Rev. Genet. 18, 411–424. 10.1038/nrg.2017.26 28502977

[B58] Wang Z. Lucas F. Qiu P. Liu Y. (2014). Improving the sensitivity of sample clustering by leveraging gene co-expression networks in variable selection. BMC Bioinforma. 15, 153. 10.1186/1471-2105-15-153 PMC4035826 24885641

[B59] Warnecke T. Hurst L. (2007). Evidence for a trade-off between translational efficiency and splicing regulation in determining synonymous codon usage in *Drosophila melanogaster* . Mol. Biol. Evol. 24, 2755–2762. 10.1093/molbev/msm210 17905999

[B60] Wendel J. F. (2000). Genome evolution in polyploids. Plant Mol. Biol. 42, 225–249. 10.1023/a:1006392424384 10688139

[B61] Wendel J. Lisch D. Hu G. Mason A. (2018). The long and short of doubling down: Polyploidy, epigenetics, and the temporal dynamics of genome fractionation. Curr. Opin. Genet. Dev. 49, 1–7. 10.1016/j.gde.2018.01.004 29438956

[B62] Xing Y. Lee C. (2006). Alternative splicing and RNA selection pressure — Evolutionary consequences for eukaryotic genomes. Nat. Rev. Genet. 7, 499–509. 10.1038/nrg1896 16770337

[B63] Yang X. Yu H. Sun W. Ding L. Li J. Cheema J. (2021). Wheat *in vivo* RNA structure landscape reveals a prevalent role of RNA structure in modulating translational subgenome expression asymmetry. Genome Biol. 22, 326. 10.1186/s13059-021-02549-y 34847934 PMC8638558

[B64] Yuan J. Jiao W. Liu Y. Ye W. Wang X. Liu B. (2020). Dynamic and reversible DNA methylation changes induced by genome separation and merger of polyploid wheat. BMC Biol. 18, 171. 10.1186/s12915-020-00909-x 33218336 PMC7679994

[B65] Zhang G. Hubalewska M. Ignatova Z. (2009). Transient ribosomal attenuation coordinates protein synthesis and co-translational folding. Nat. Struct. Mol. Biol. 16, 274–280. 10.1038/nsmb.1554 19198590

[B66] Zhang W. Sun X. Yuan H. Araki H. Wang J. Tian D. (2008). The pattern of insertion/deletion polymorphism in *Arabidopsis thaliana* . Mol. Genet. Genomics 280, 351–361. 10.1007/s00438-008-0370-1 18690477

[B67] Zhao M. Zhang B. Lisch D. Ma J. (2017). Patterns and consequences of subgenome differentiation provide insights into the nature of paleopolyploidy in plants. Plant Cell 29, 2974–2994. 10.1105/tpc.17.00595 29180596 PMC5757279

[B68] Zohary D. Feldman M. (1962). Hybridization between amphidiploids and the evolution of polyploids in the wheat (*Aegilops-Triticum*) group. Evolution 16, 44–61. 10.2307/2406265

