# Supplementary material for: Alteration of synonymous codon usage bias accompanies polyploidization in wheat
Source: Front Genet. 2022 Oct 14;13:979902. doi: 10.3389/fgene.2022.979902 (PMC9614214; doi:10.3389/fgene.2022.979902)
Supplement: Supplementary file 1 [file DataSheet1.PDF]

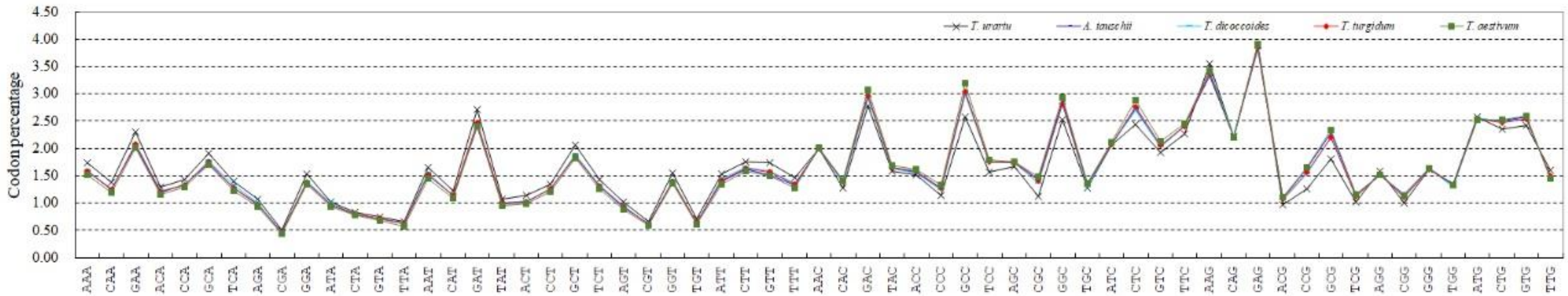

Figure S1. Frequency of all 61 codons specifying amino acids in the *Triticum/Aegilops* spp. genomes. The frequency was defined as the number of instances of each codon divided by the total number of these 61 codons.

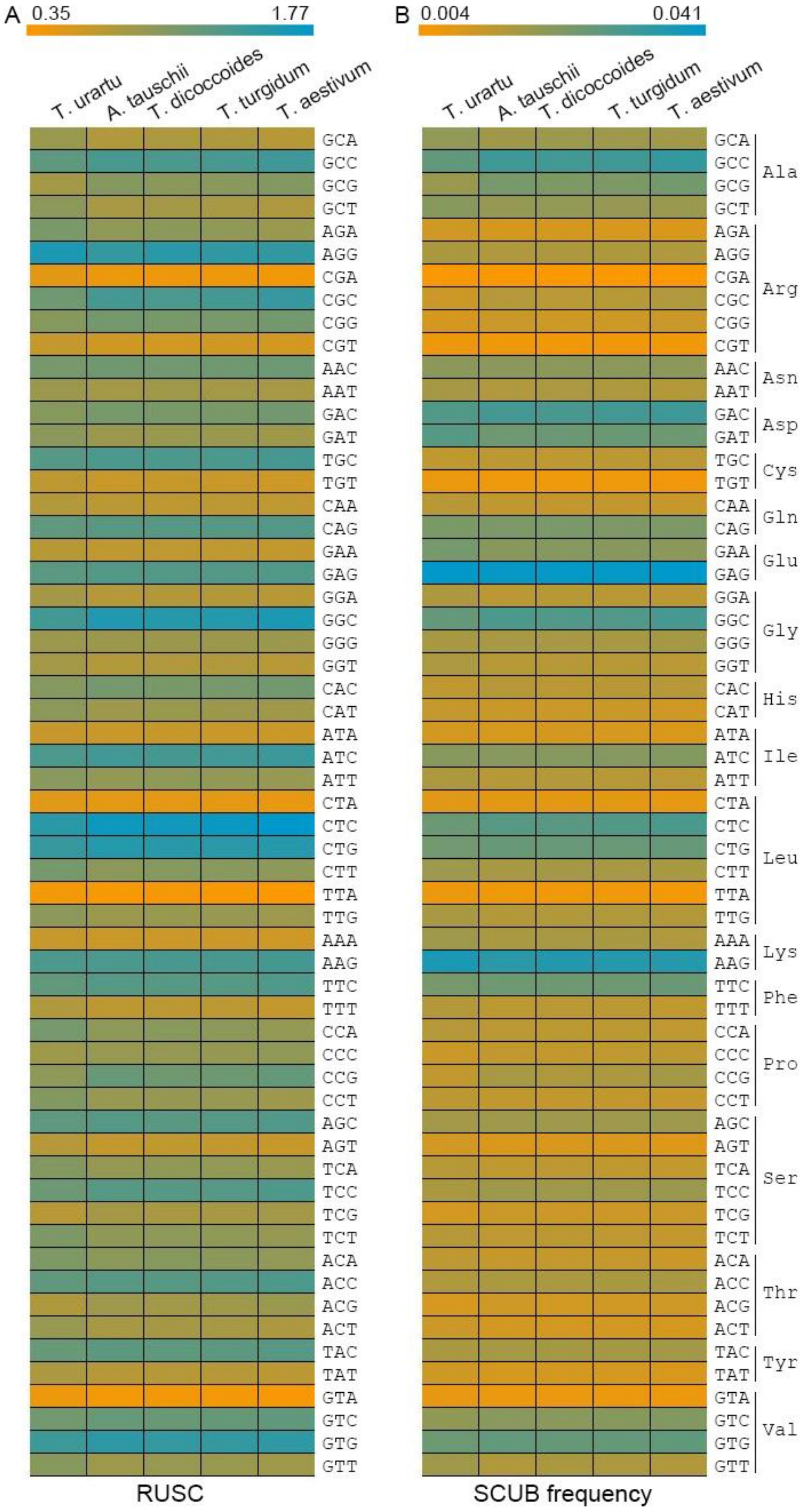

Figure S2. Comparison between RSCU values (A) and SCUB frequencies (B) for 59 SCs specifying 18 amino acids. RSCU values were calculated with CodonW software. SCUB frequencies were calculated as the ratio between the number of each SC and the total number of SCs.

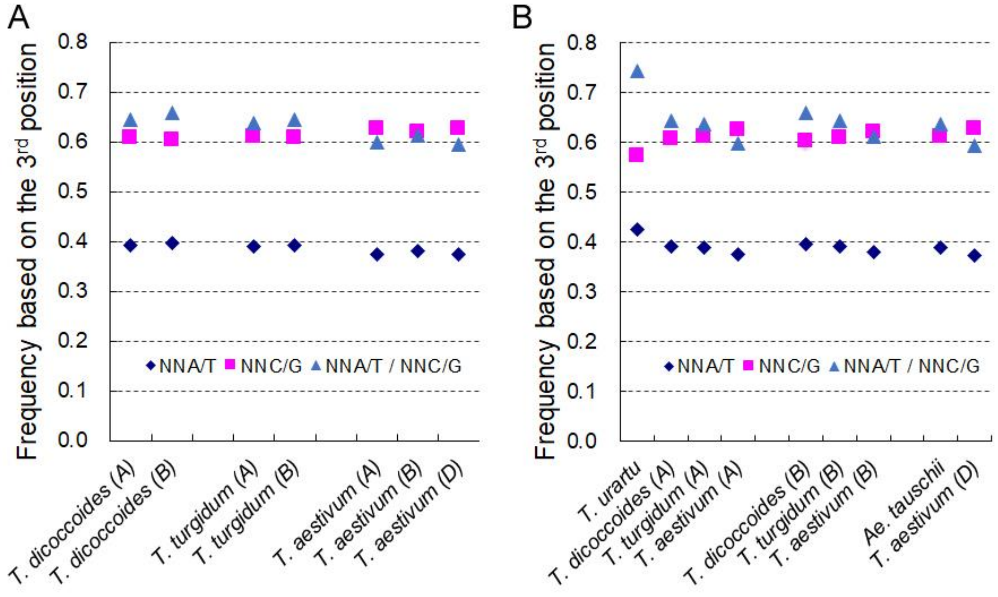

Figure S3. SCUB of subgenomes is heterogeneous between hexaploid wheat and its progenitors. (A) Frequencies of NNA/T and NNC/G codons of subgenomes in tetraploid and hexaploid wheat. (B) Frequencies of NNA/T and NNC/G codons of A, B or D subgenome among hexaploid wheat and its progenitors. NNA/T and NNC/G: SCs with A and T or C and G as the final base, respectively; N denotes any base. The frequency was calculated as the ratio between the number of all SCs ending with A and T or C and G and the total number of SCs.

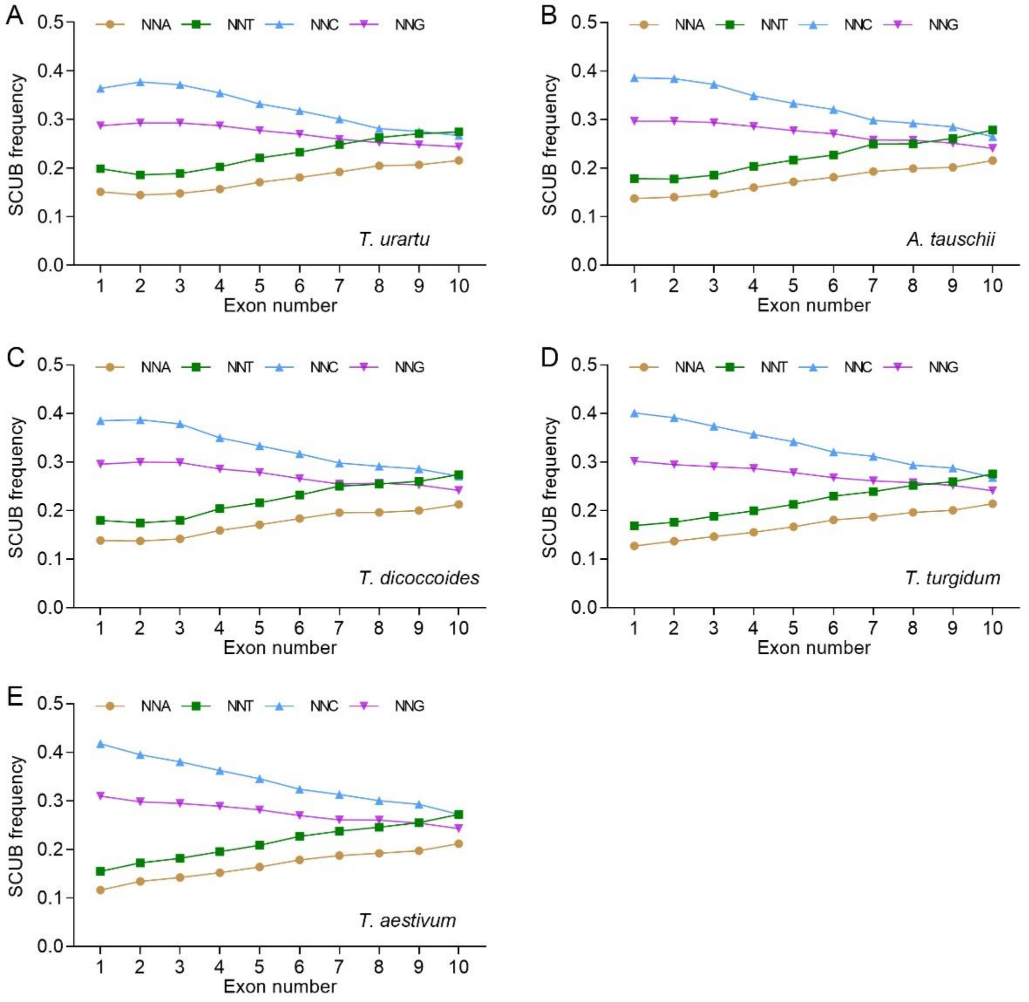

Figure S4. SCUB frequencies in genes with up to nine exons. SCUB frequencies in the A subgenome diploid progenitor (A), the D subgenome diploid progenitor (B), the AB genome from wild tetraploid wheat (C), the AB genome of domestic tetraploid wheat (D), and hexaploid wheat (E). NNA, NNT, NNC and NNG: SCs with A, T, C and G as the final base, respectively; N denotes any base.

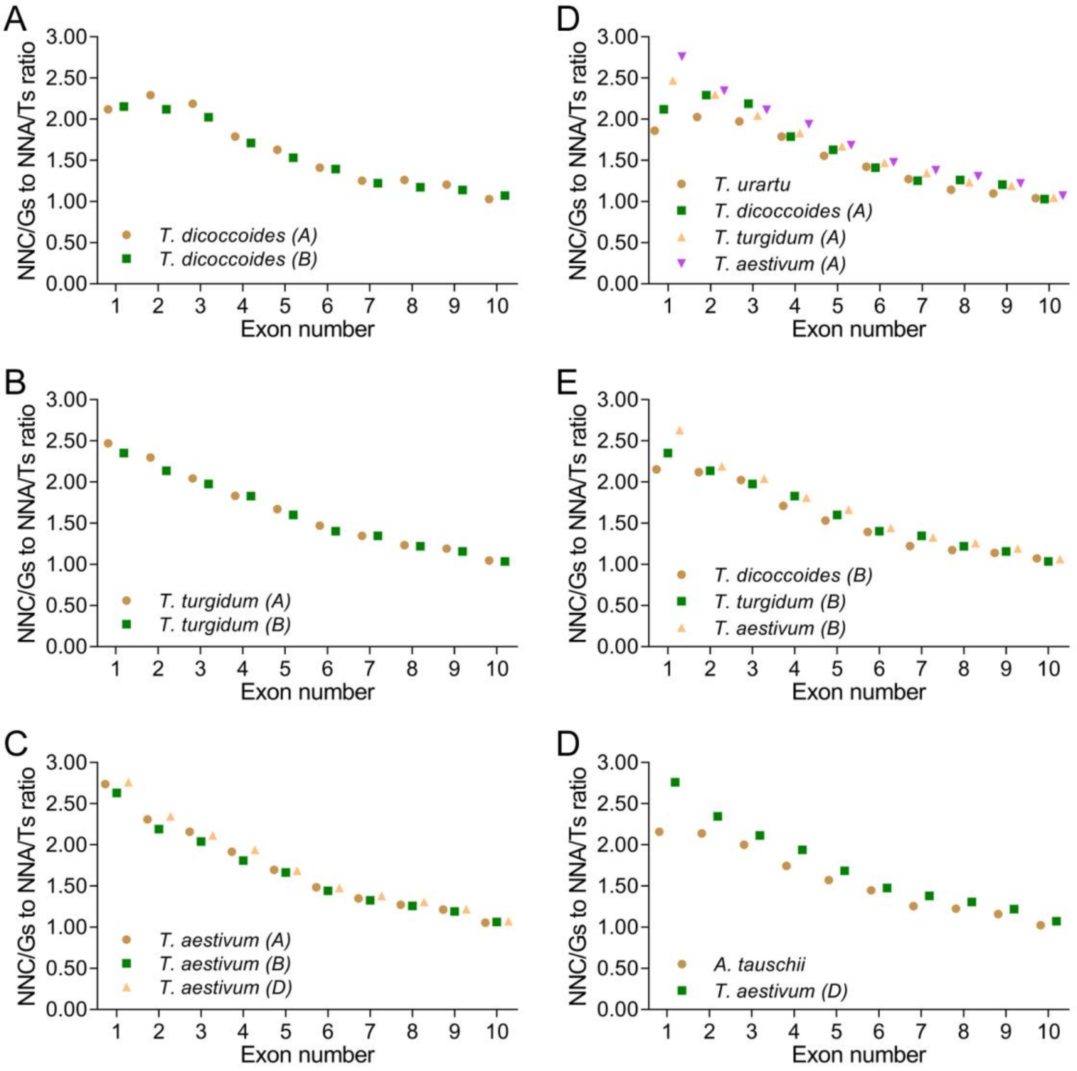

Figure S5. Influence of the number of introns on SCUB at subgenome level. (A-C) Ratios between A/T-ending SCs and C/G-ending SCs (NNA/Ts and NNC/Gs) in genes with up to nine introns among subgenomes in tetraploid and hexaploid wheat. (D-F) Ratios between NNA/Ts and NNC/Gs in genes with up to nine introns in A, B or D subgenomes among hexaploid wheat and its progenitors. N denotes any base.

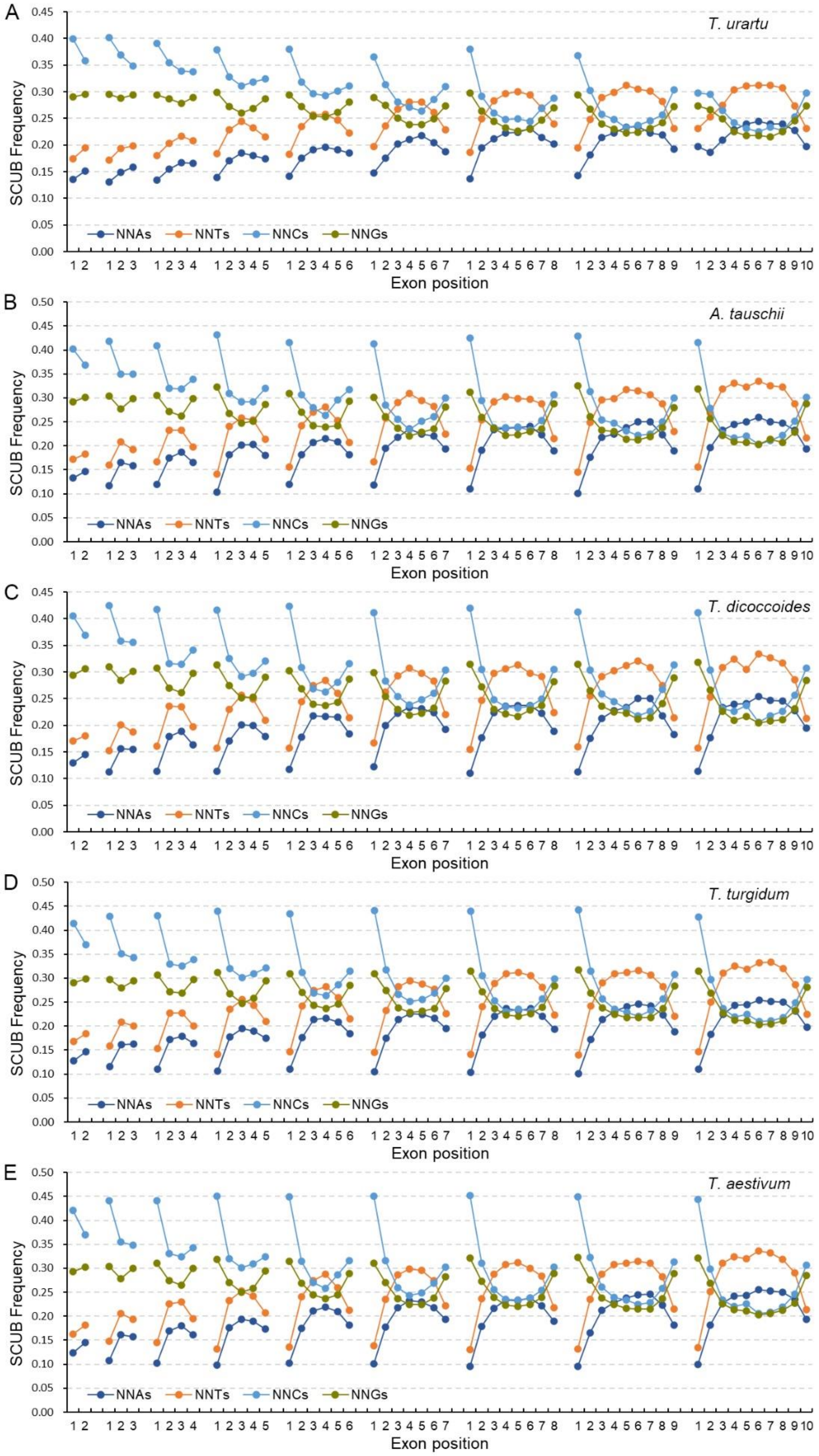

Figure S6. SCUB frequencies in different exons of genes with up to ten exons. SCUB frequencies in the A subgenome diploid progenitor (A), the D subgenome diploid progenitor (B), the AB genome from wild tetraploid wheat (C), the AB genome of domestic tetraploid wheat (D), and hexaploid wheat (E). NNA, NNT, NNC and NNG: SCs with A, T, C and G as the final base, respectively; N denotes any base.

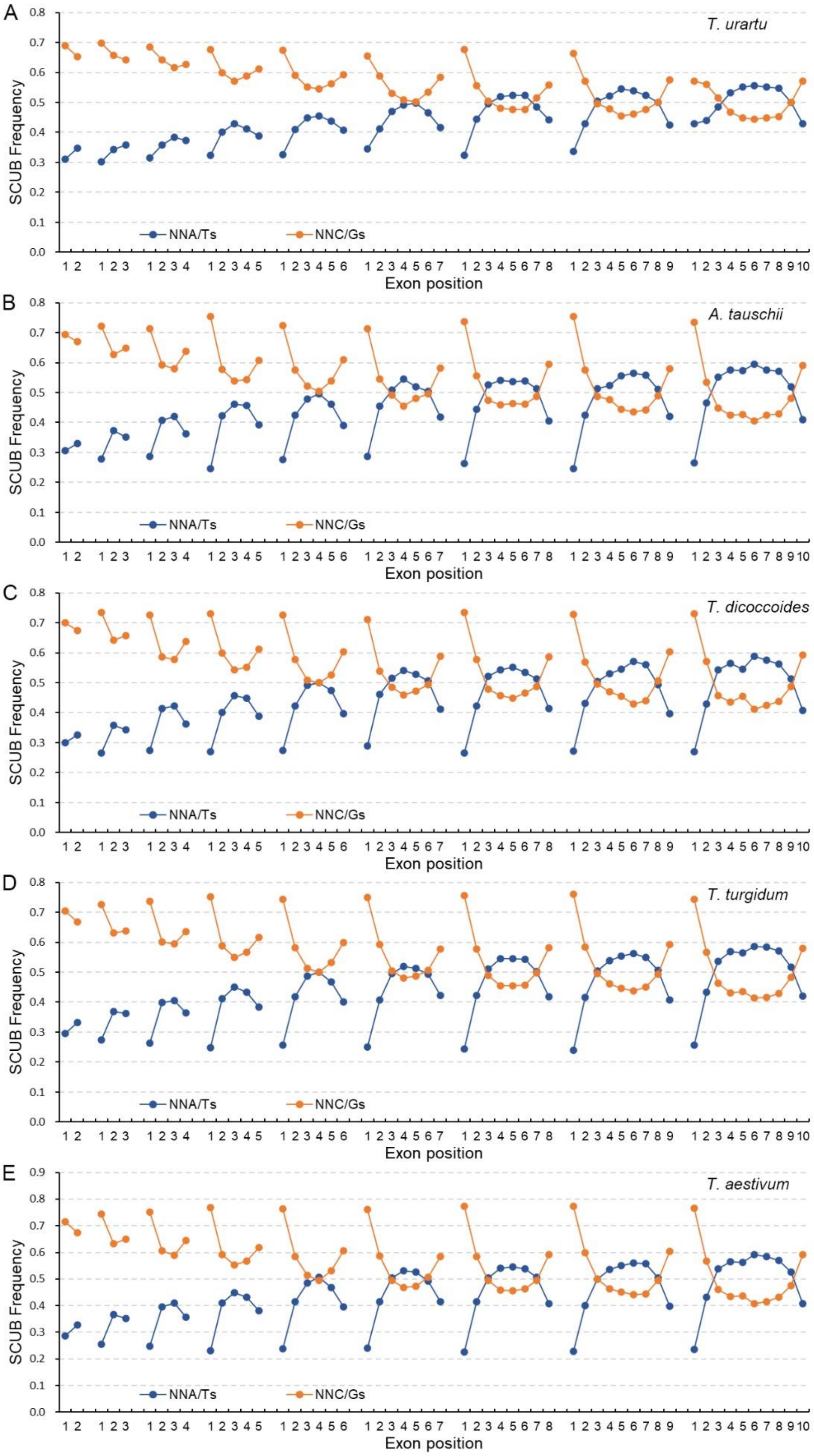

Figure S7. The frequencies of SCs ending in A/T or C/G in different exons of genes with up to ten exons. SCUB frequencies in the A subgenome diploid progenitor (A), the D subgenome diploid progenitor (B), the AB genome of wild tetraploid wheat (C), the AB genome from domestic tetraploid wheat (D), and hexaploid wheat (E). NNA/Ts: SCs with A or T as the final base; NNC/Gs: SCs with C or G as the final base; N denotes any base.

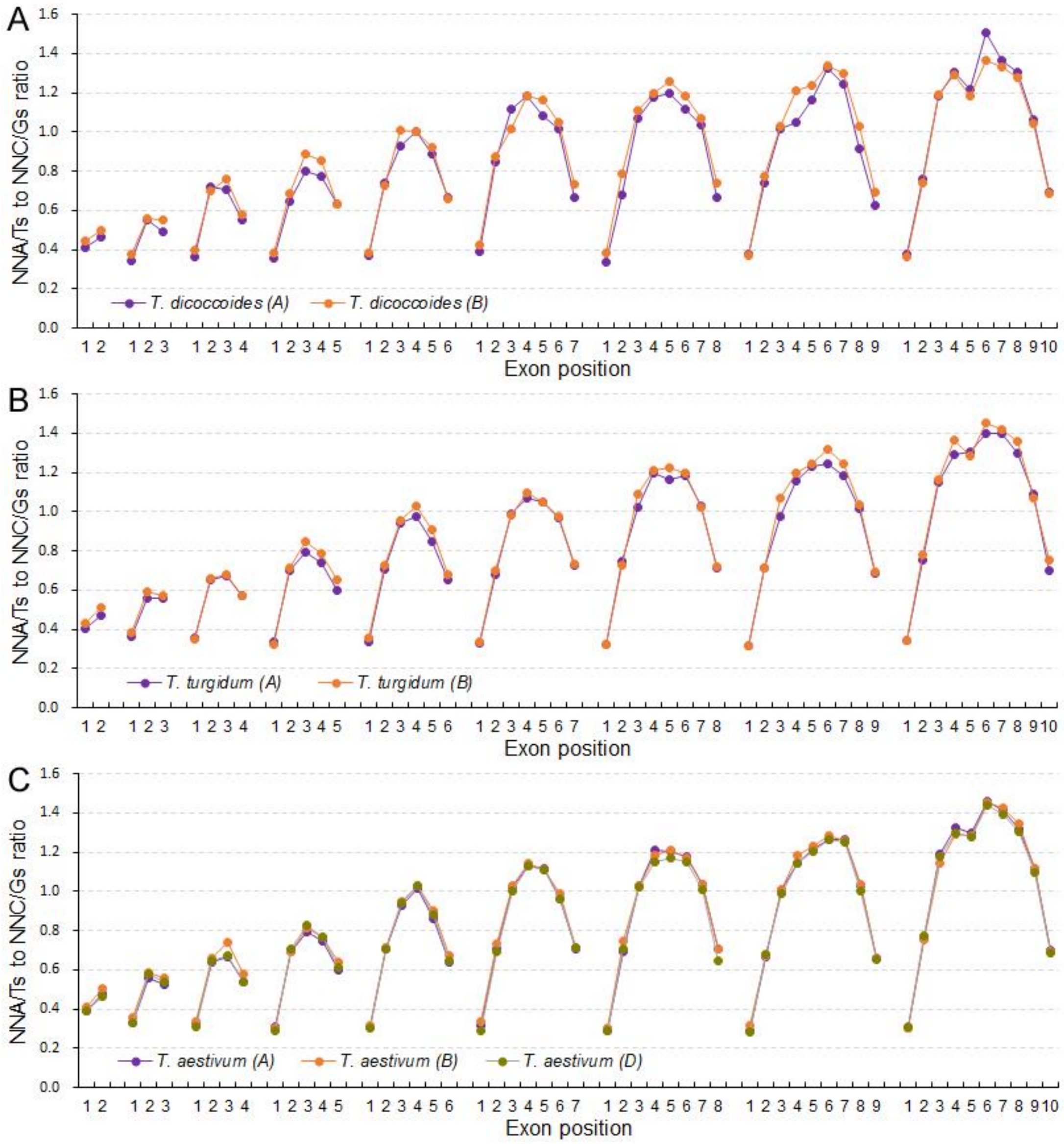

Figure S8. Ratios between A/T-ending SCs and C/G-ending SCs (NNA/Ts and NNC/Gs) as a function of exon position in genes with one to nine introns among the subgenomes in tetraploid (A, B) and hexaploid (C) wheat. N denotes any base.

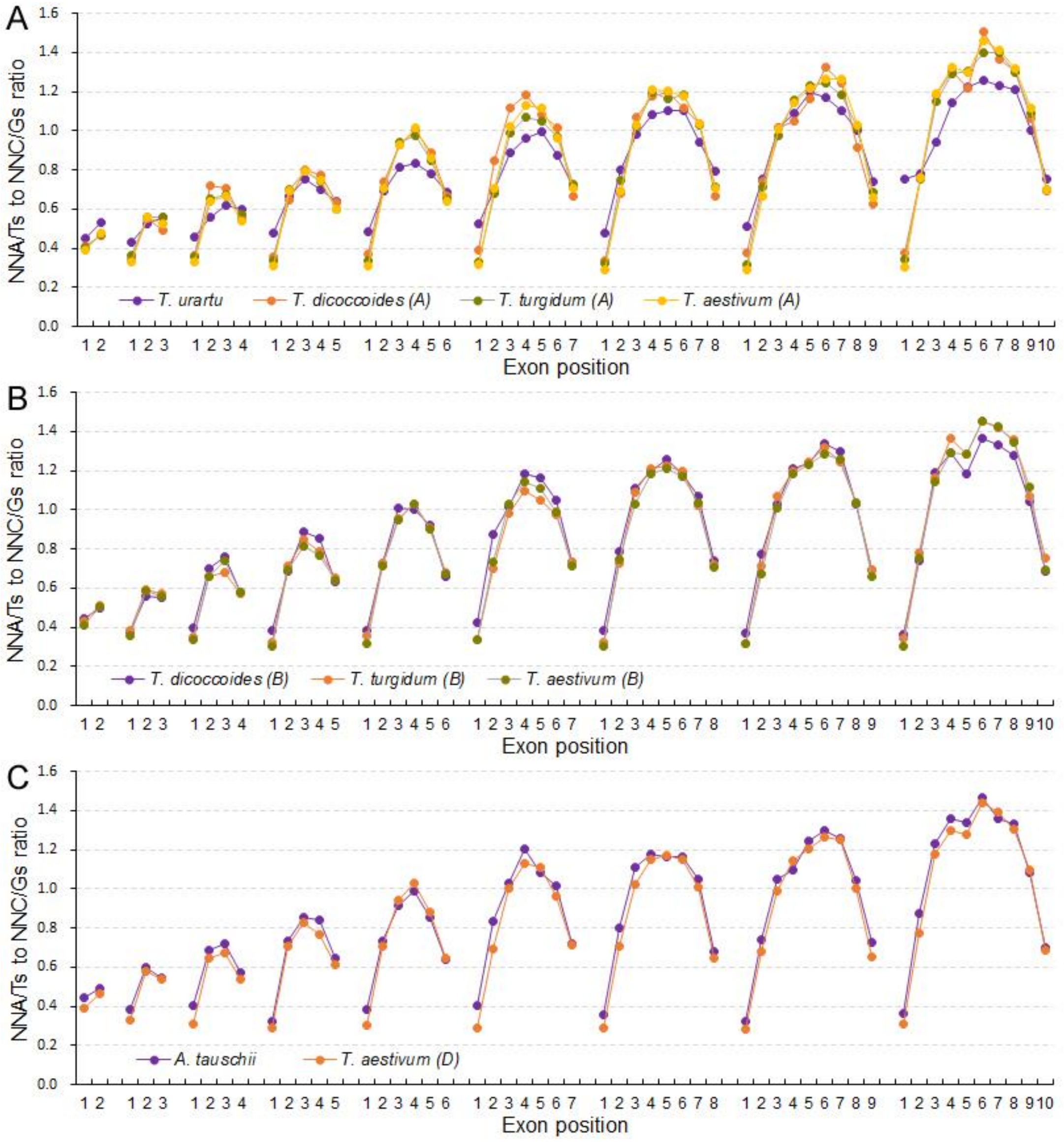

Figure S9. Ratios between A/T-ending SCs and C/G-ending SCs (NNA/Ts and NNC/Gs) as a function of exon position in genes with one to nine introns among hexaploid wheat and its progeinitors at subgenome level. (A) A subgenome comparison. (B) B subgenome comparison. (C) D subgenome comparison. N denotes any base.

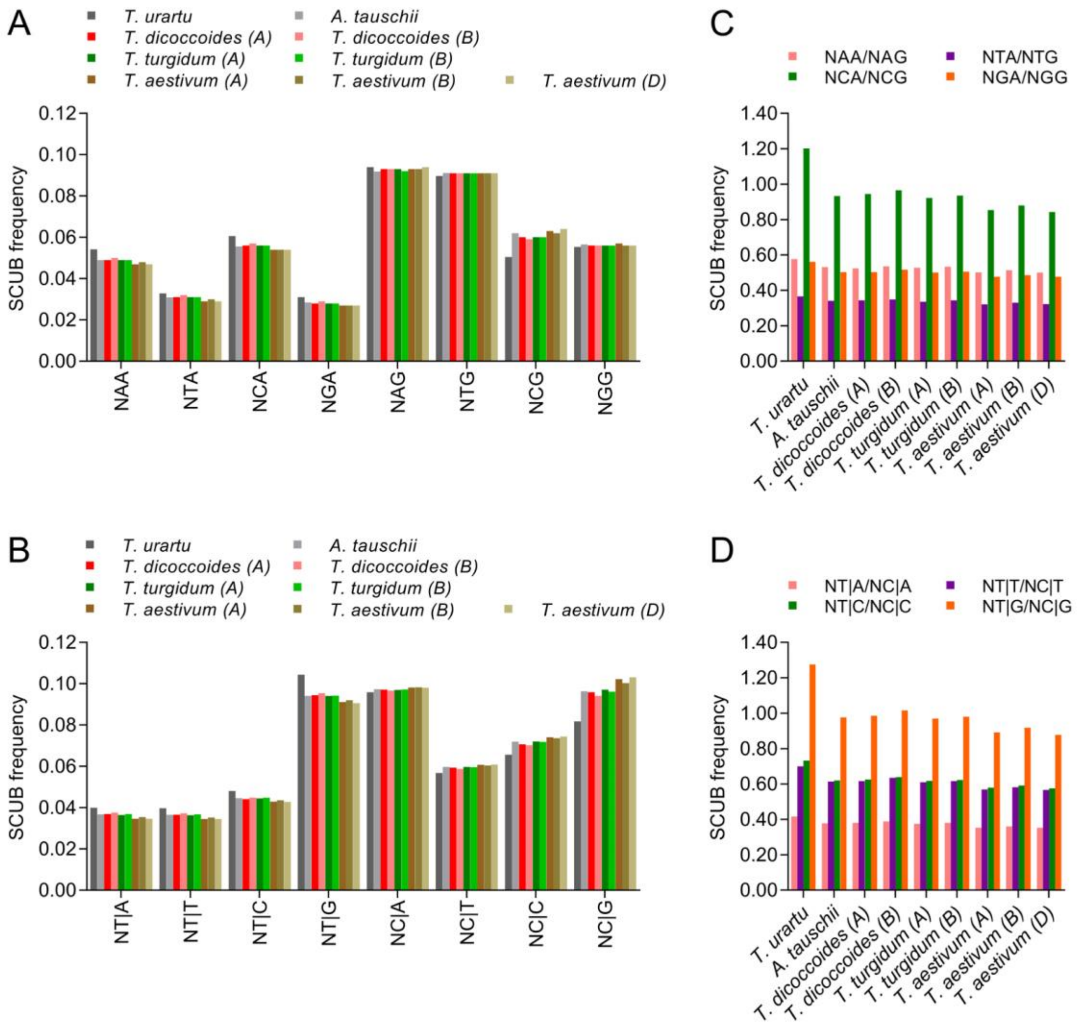

Figure S10. Association between SCUB and DNA methylation-driven conversion of cytosines to thymines at subgenome level. (A) SCUB frequencies of NNA and NNG indicating the effect of the second nucleotide position of codons on the conversion of C to T at the third position on the antisense strand based on the subgenome. (B) SCUB frequencies of NT|N and NC|N indicating the effect of the first nucleotide position of the next codon on the conversion of C to T at the third position of the previous codon on the sense strand based on the subgenome. (C) Ratios between NNA and NNG codon frequencies based on the subgenome. (D) Ratios between NT|N and NC|N triplets based on the subgenome. NNA and NNG: SCs with A and G as the final bases and any base at the second position; N denotes any base. NT|N and NC|N: SCs with C and T as the final base of the previous codon and any base at the first position of the next codon.

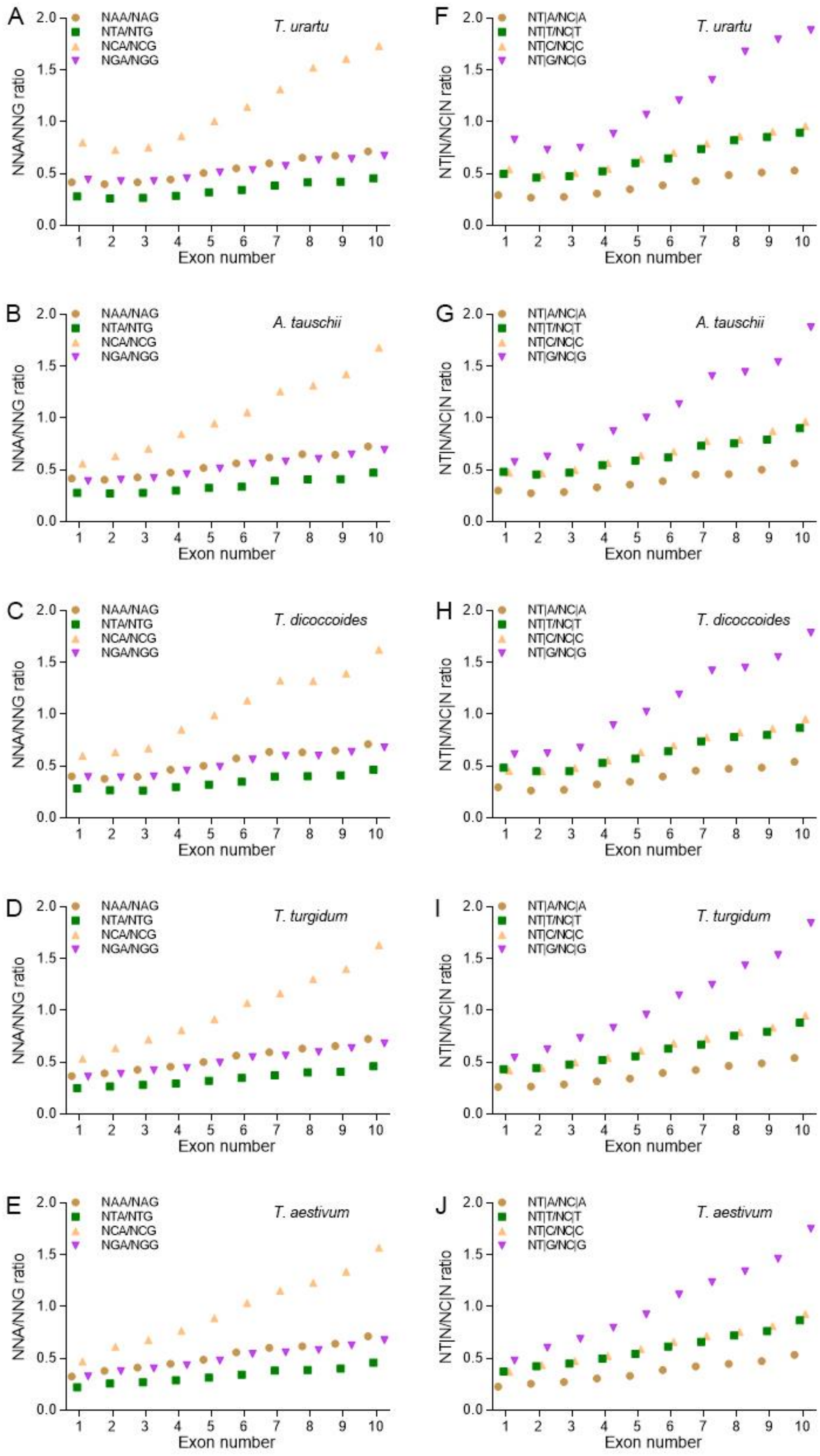

Figure S11. Association between DNA methylation and SCUB in genes with up to nine introns. (A–E): Effect of the second nucleotide on the bias seen for A or G at the third position. (F–J): Effect of the first nucleotide of the next codon on the bias seen for T or C at the third position.

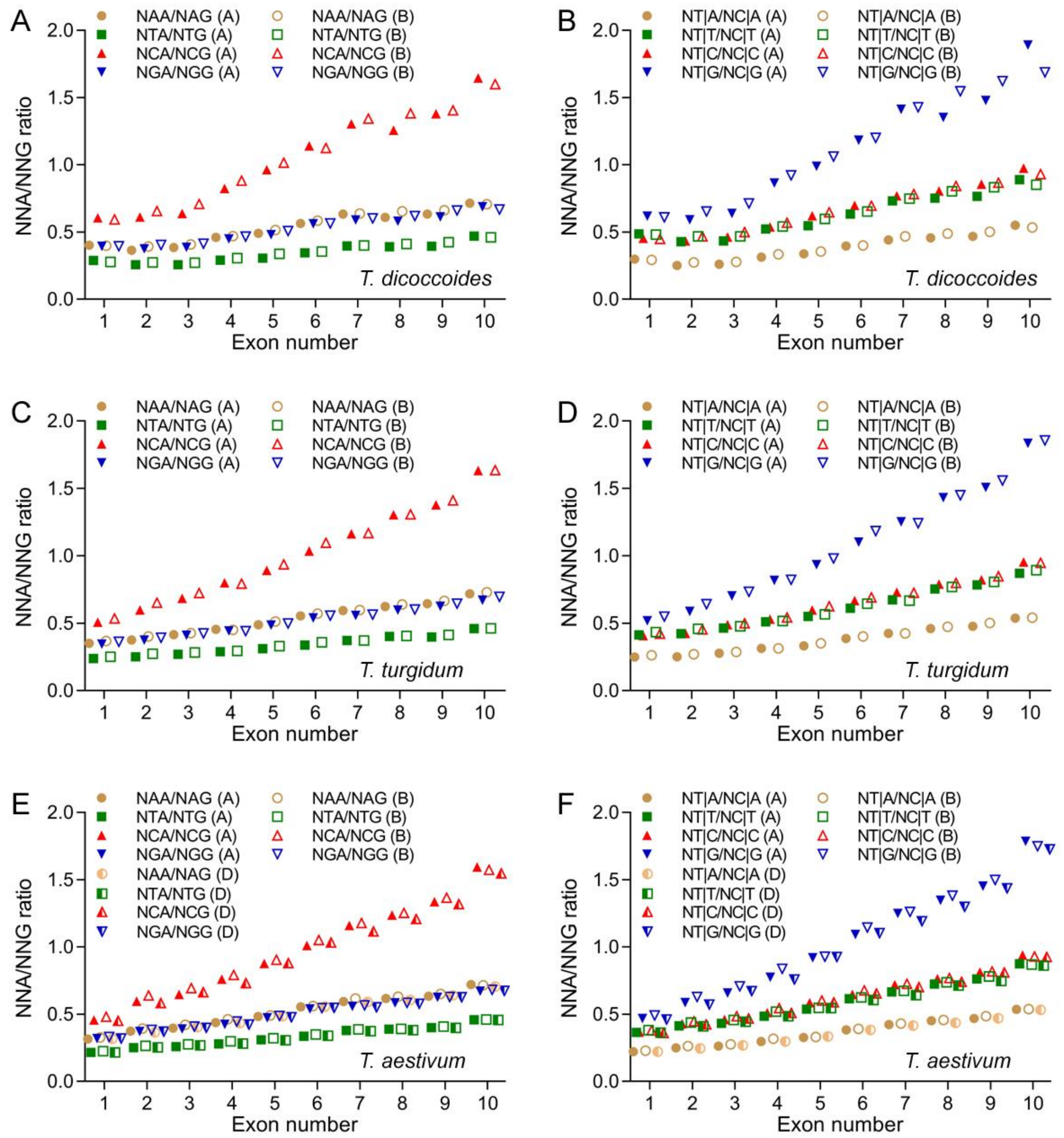

Figure S12. Association between DNA methylation and SCUB in genes with up to nine introns among the subgenomes in tetraploid and hexaploid wheat. (A, C, E): Effect of the second nucleotide on the bias seen for A or G at the third position. (B, D, F): Effect of the first nucleotide of the next codon on the bias seen for T or C at the third position. (A), (B) and (D) in the panels mean A, B and D subgenomes.

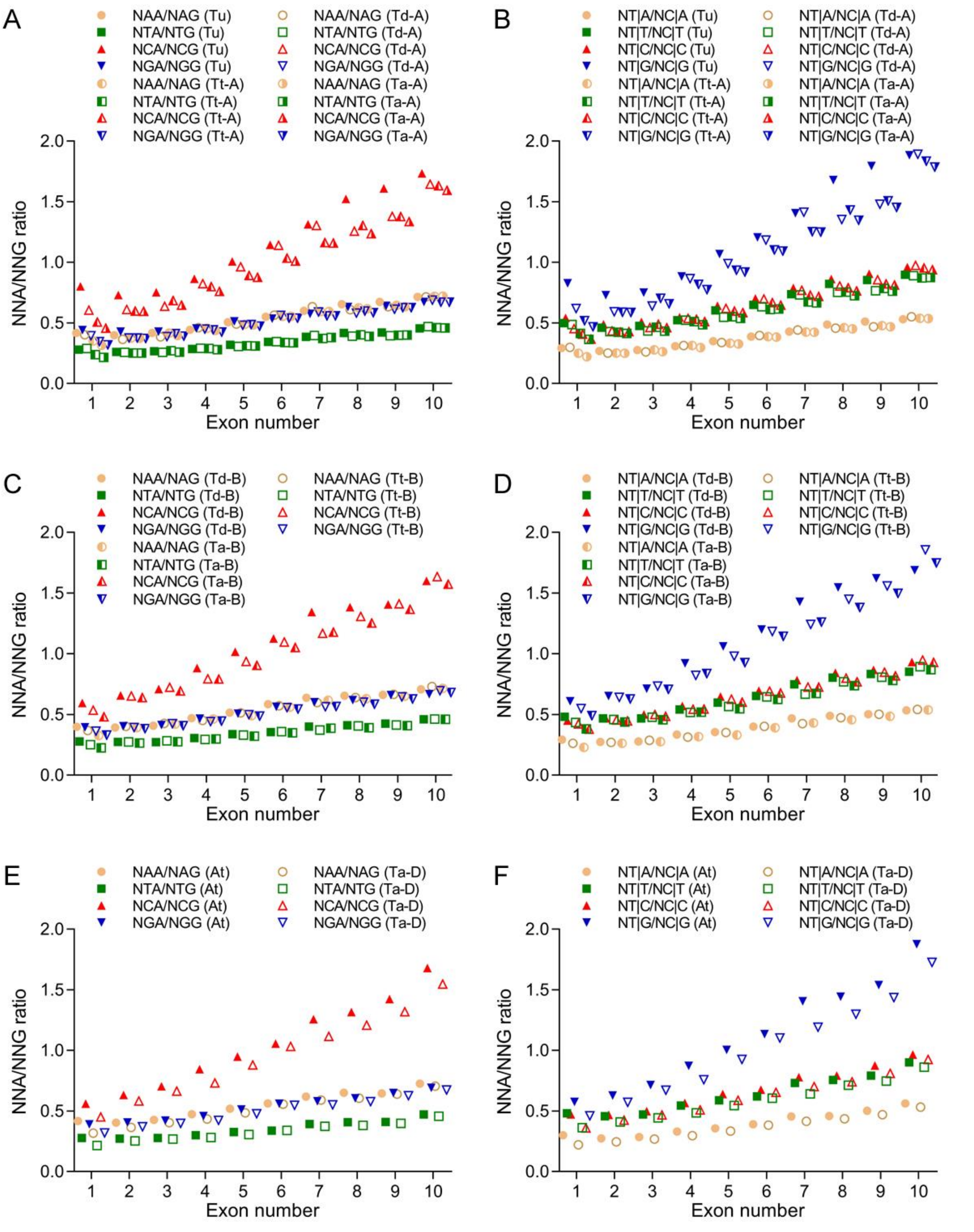

Figure S13. Association between DNA methylation and SCUB in genes with up to nine introns among hexaploid wheat and its progenitors at the subgenome level. (A, C, E): Effect of the second nucleotide on the bias seen for A or G at the third position. (B, D, F): Effect of the first nucleotide of the next codon on the bias seen for T or C at the third position. Tu: *T. urartu*; At: *Aegilops tauschii*; Td: *T. dicoccoides*; Tt: *T. turgidum*; Ta: *Triticum aestivum*. A, B and D in the panels mean A, B and D subgenomes.

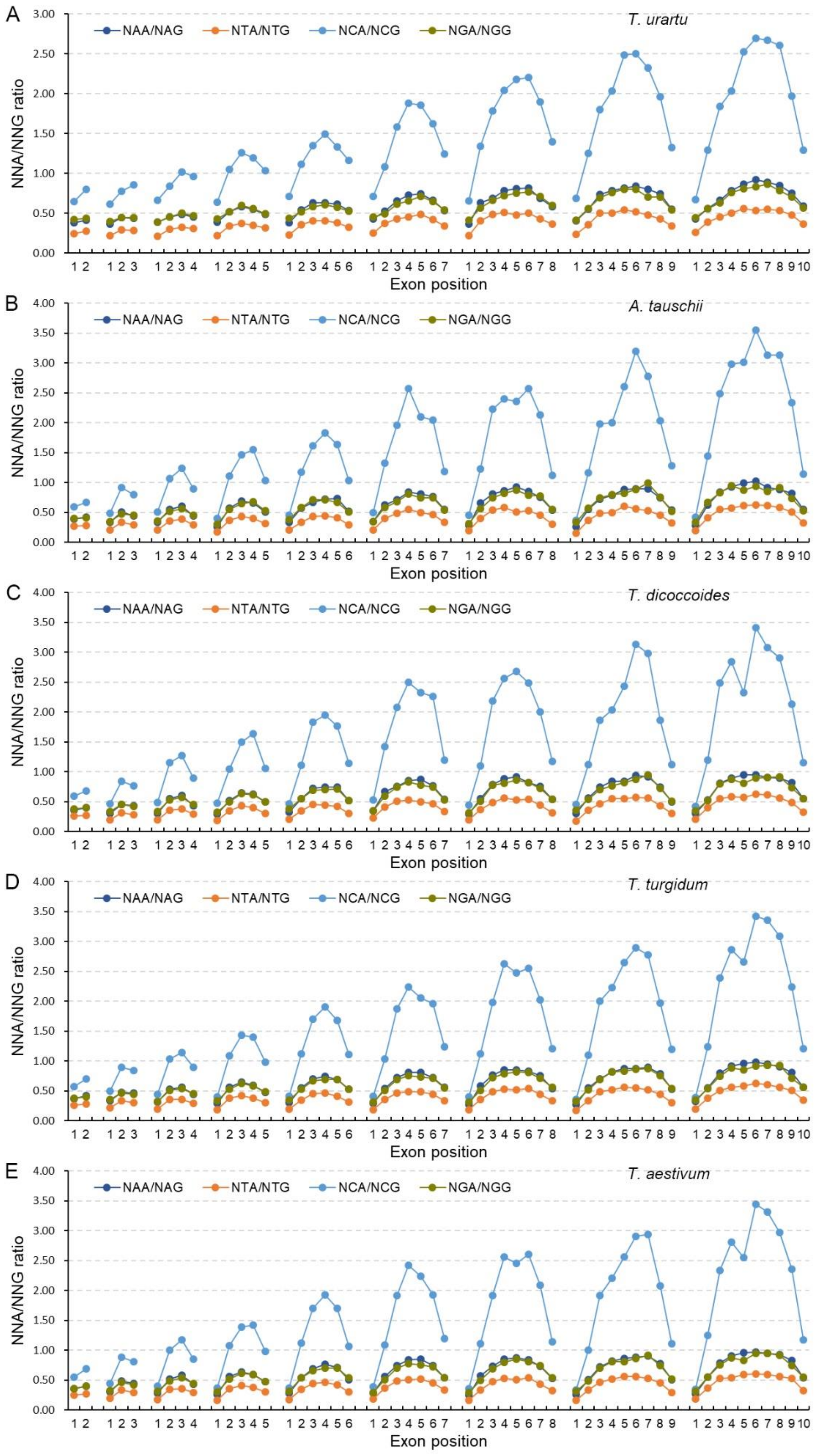

Figure S14. Association between DNA methylation and SCUB in different exons of genes with up to ten exons, with a focus on the effect of the nucleotide at the second position of a codon on the bias seen for A or G at the third position.

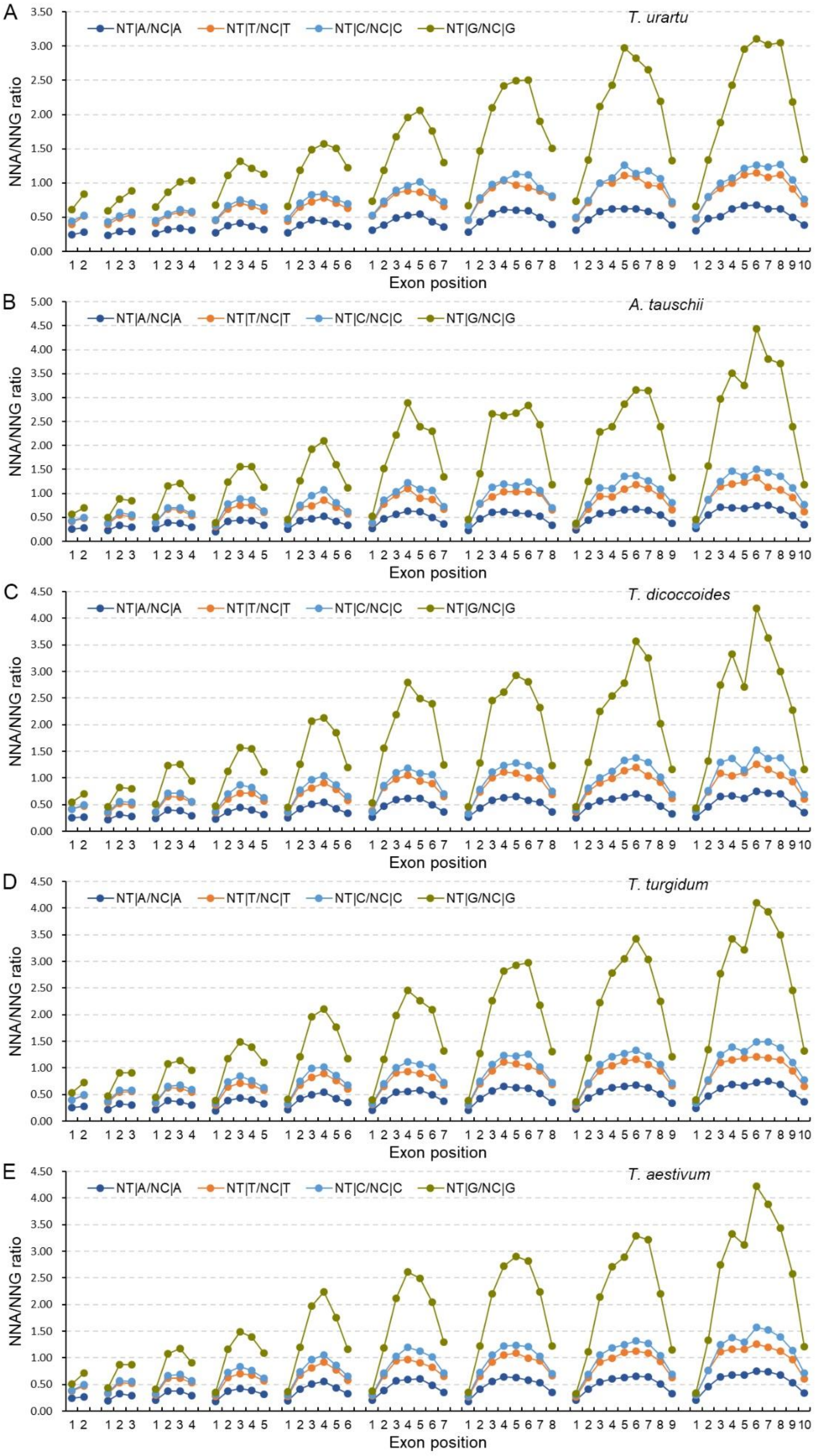

Figure S15. Association between DNA methylation and SCUB in different exons of genes with up to nine introns, with a focus on the effect of the nucleotide at the first position in the next codon on the bias seen for T or C at the third position of the previous codon.

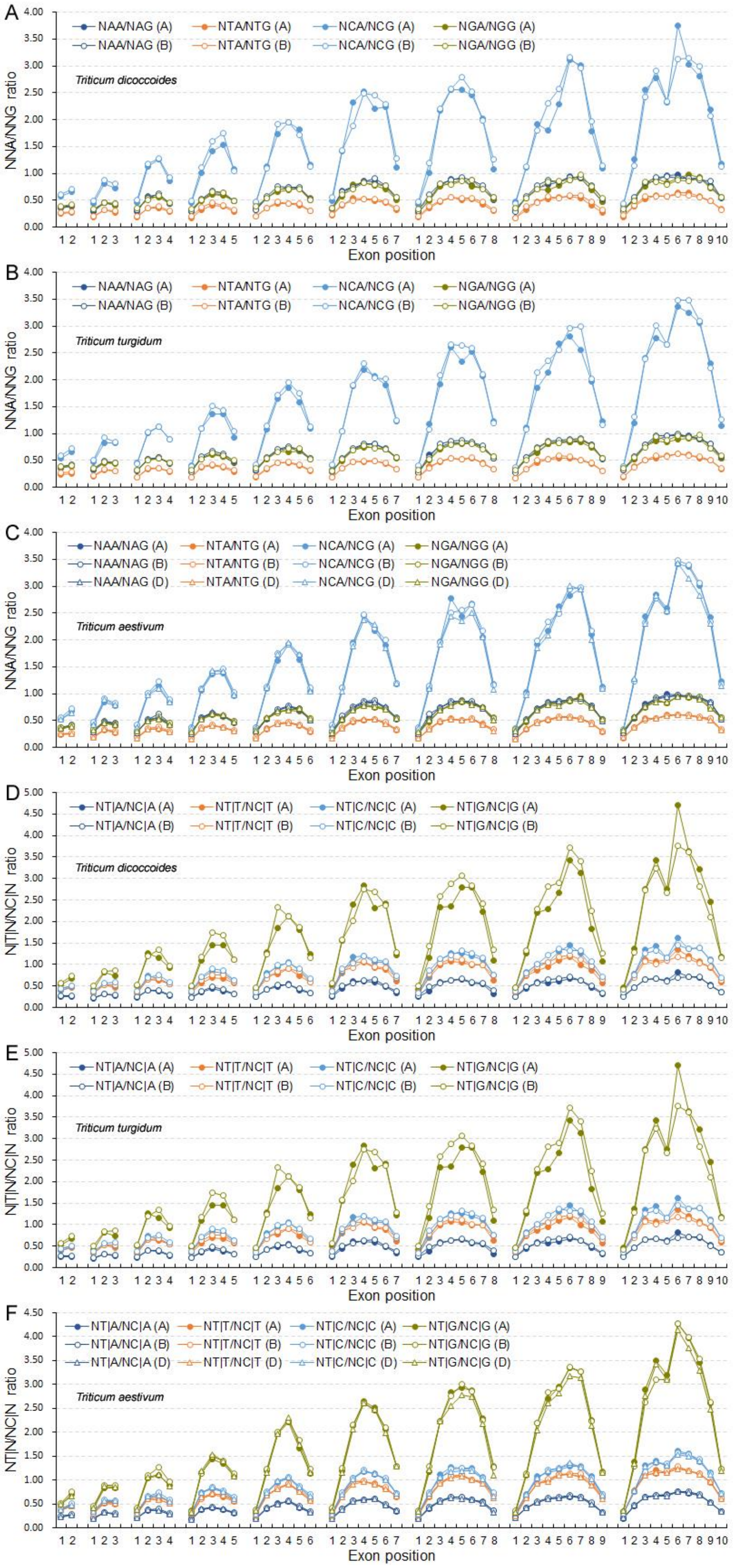

Figure S16. Association between DNA methylation and SCUB in different exons of genes with up to nine introns among the subgenomes in tetraploid and hexaploid wheat. The comparison was conducted with a focus on the effect of the nucleotide at the second position of a codon on the bias seen for A or G at the third position (A-C) and with a focus on the effect of the nucleotide at the first position in the next codon on the bias seen for T or C at the third position of the previous codon (D-F). A, B and D in the panels mean A, B and D subgenomes.

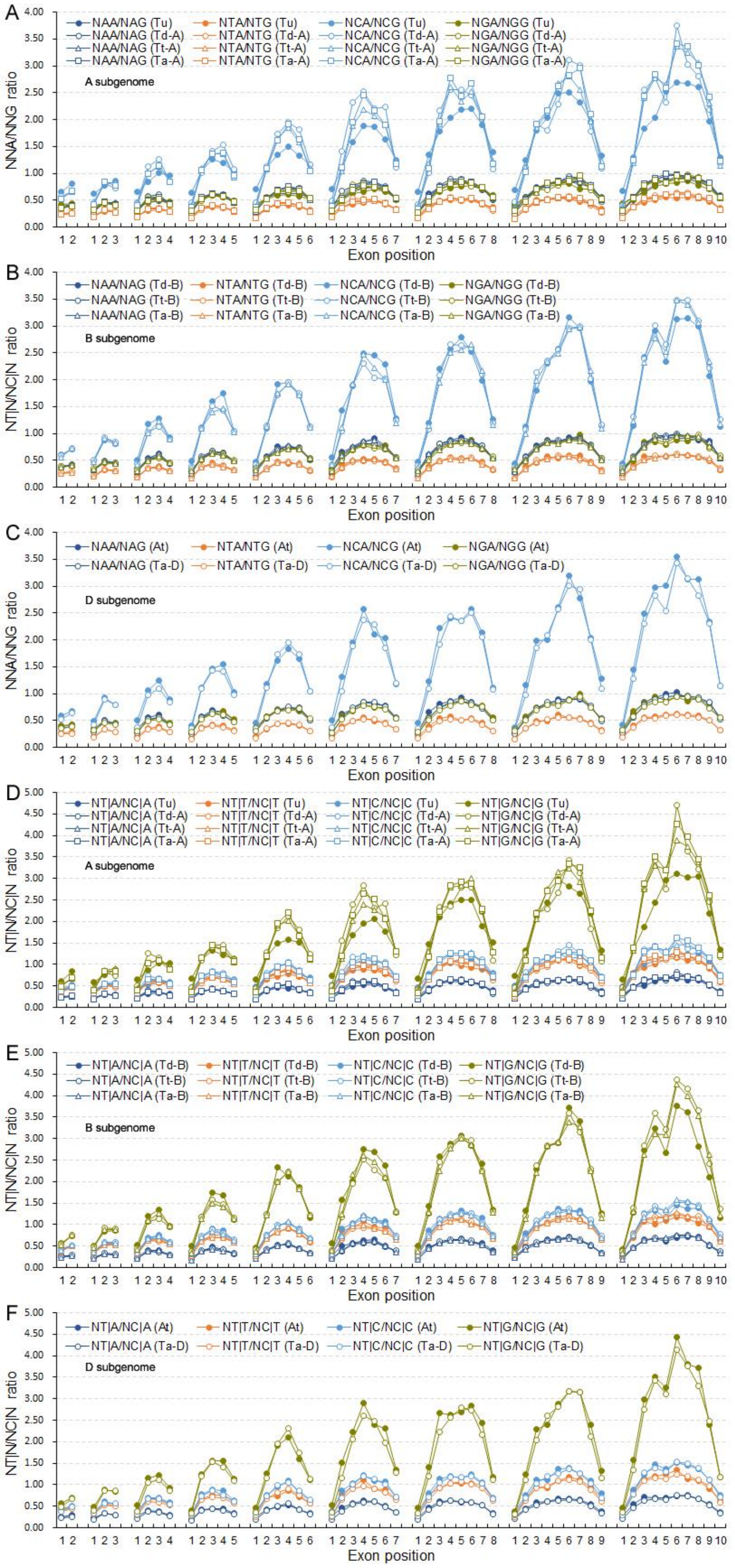

Figure S17. Association between DNA methylation and SCUB in different exons of genes with up to nine introns among hexaploid wheat and its progenitors at the subgenome level. The comparison was conducted with a focus on the effect of the nucleotide at the second position of a codon on the bias seen for A or G at the third position (A-C) and with a focus on the effect of the nucleotide at the first position in the next codon on the bias seen for T or C at the third position of the previous codon (D-F). Tu: *T. urartu*; At: *Aegilops tauschii*; Td: *T. dicoccoides*; Tt: *T. turgidum*; Ta: *Triticum aestivum*. A, B and D in the panels mean A, B and D subgenomes.

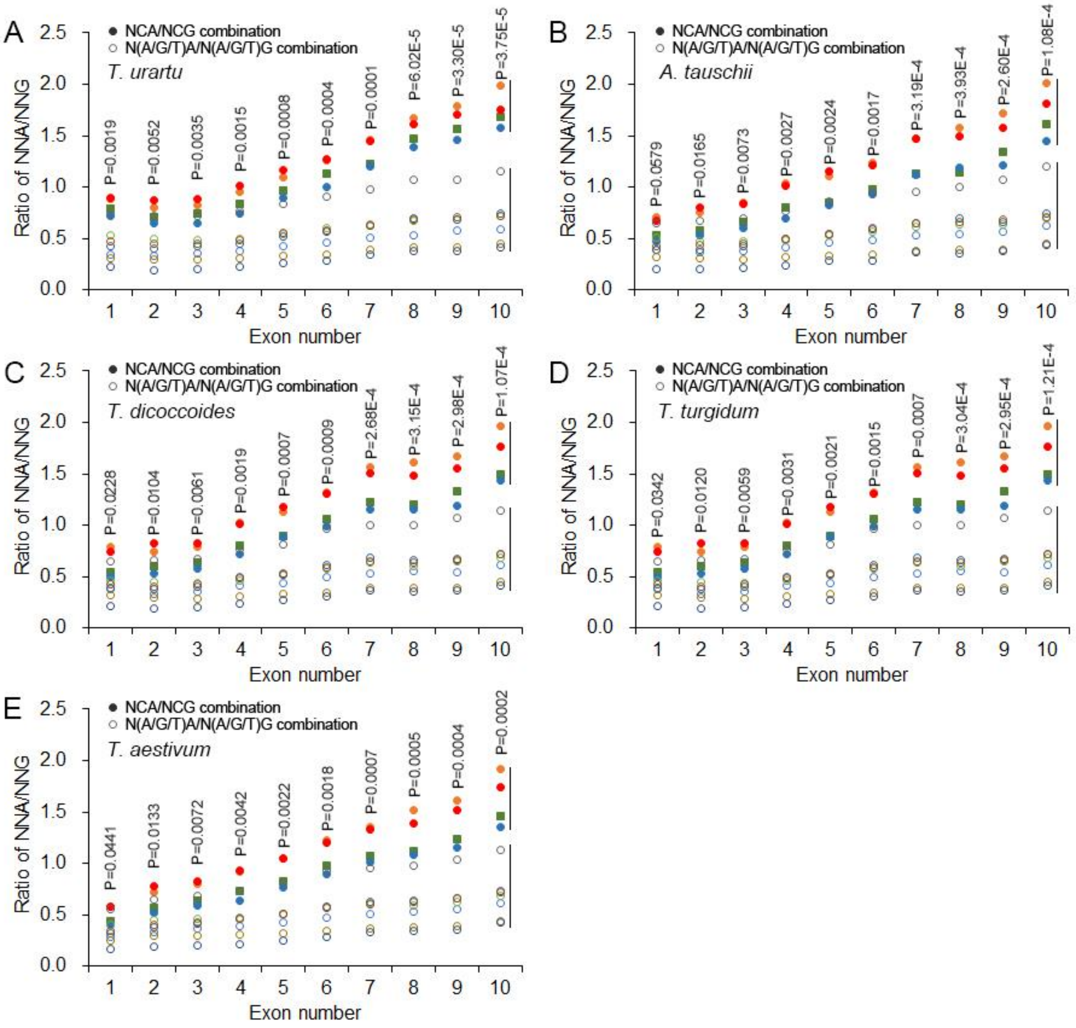

Figure S18. Ratios between A-ending SCs and G-ending SCs specifying a given amino acids in genes with up to nine introns. NCA/NCG: ratio between NCA SCs and NCG SCs, with C at the second position that specify Ala, Pro, Ser, or Thr. N(A/G/T)A / N(A/G/T)G: ratio between NNA SCs and NNG SCs with A, G and T but not C at the second position, specifying Arg, Glu, Gly, Leu, or Lys.

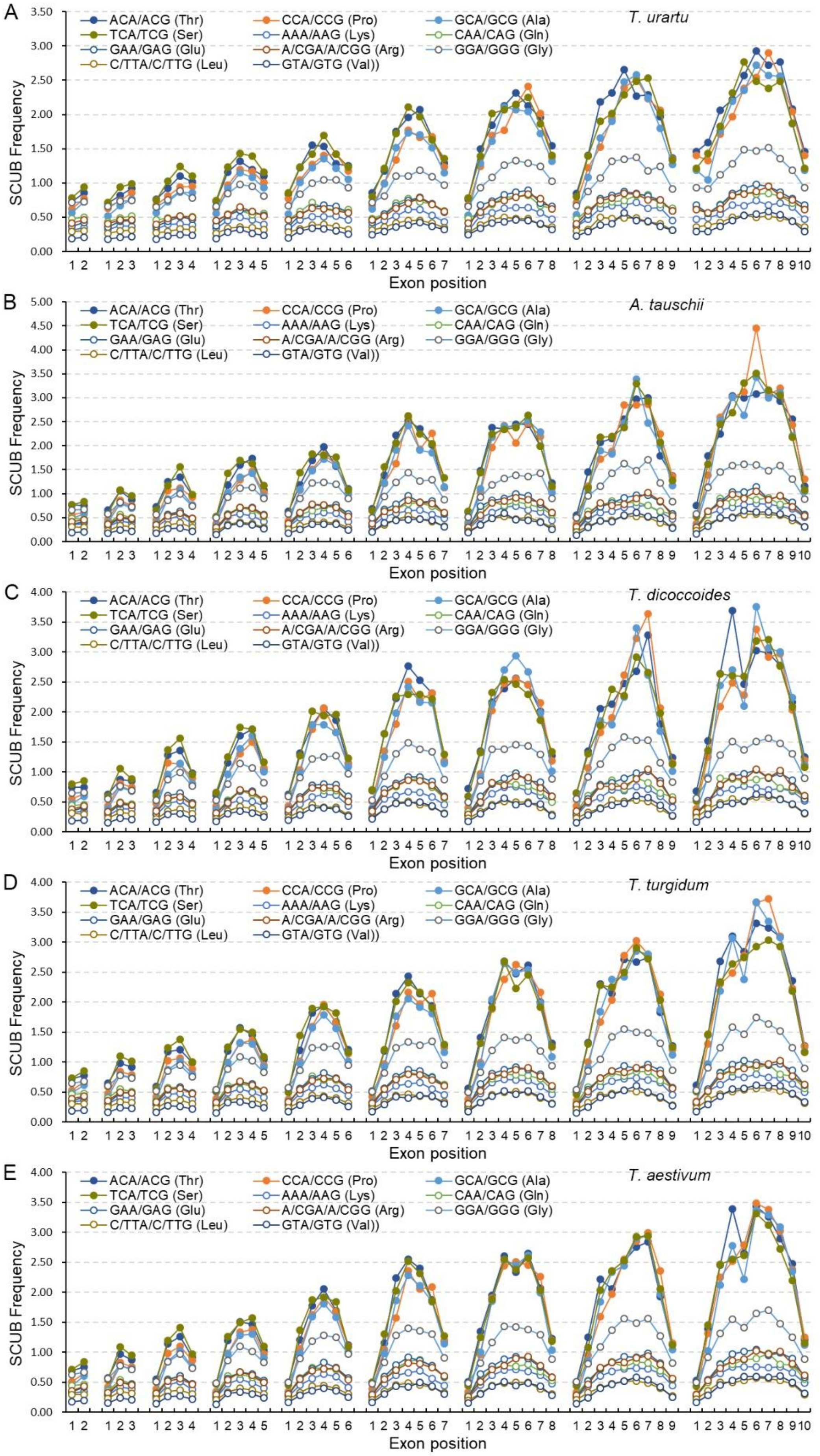

Figure S19. Ratios between A-ending SCs and G-ending SCs specifying the indicated amino acids in genes with up to nine introns as a function of exon position.

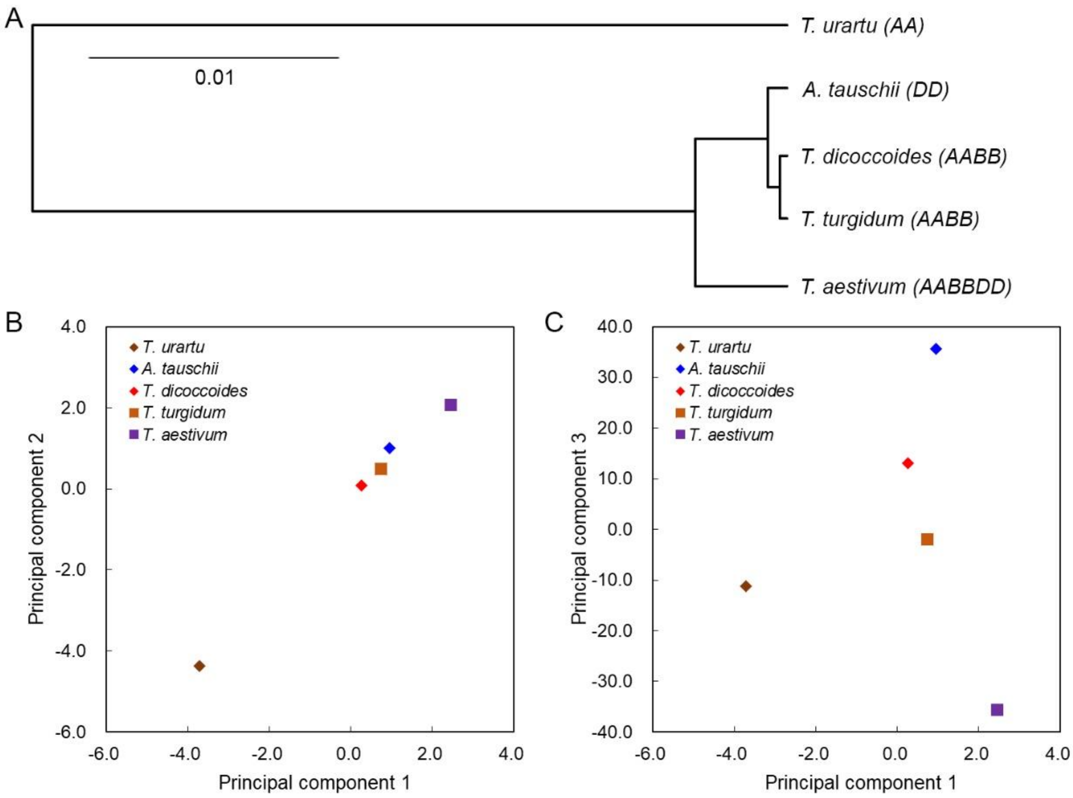

Figure S20. Clustering analysis and PCA of SCUB.

(A) Cluster tree based on SCUB frequency from a set of 59 SCs specifying 18 amino acids. (B) Two-dimensional representation of the PCA, with PC1 and PC2. (C) Two-dimensional representation of PCA results with PC1 and PC3. SCUB frequency for each SC was calculated as the ratio between the number of that SC and the number of all SCs.
